# Formononetin Protects Osteoblasts Against Oxidative Stress and Attenuates Ovariectomy-Induced Bone Loss in Association with MAPK/AP-1 and NRF2 Signaling

**DOI:** 10.3390/antiox15101348

**Published:** 2026-10-19

**Authors:** Shuhua Liu, Ruolin Wang, Chenhaoyu Liu, Jiachun Huang, Xiaoyun Li, Haomin Qian, Hongxing Huang, Lei Wan

**Affiliations:** 1 Guangzhou University of Chinese Medicine, Guangzhou 510405, China; 20201120464@stu.gzucm.edu.cn (S.L.);; 2 Shenzhen Hospital (Futian) of Guangzhou University of Chinese Medicine, Shenzhen 518034, China; 3 The Third Affiliated Hospital of Guangzhou University of Chinese Medicine, Guangzhou 510378, China

**Keywords:** formononetin, postmenopausal osteoporosis, oxidative stress, osteoblast, osteogenic differentiation, MAPK/AP-1 signaling pathway

## Abstract

Postmenopausal osteoporosis (PMOP) is associated with estrogen deficiency and oxidative stress (OS), which impair osteoblast survival and osteogenic function. Formononetin (FMN) is a phytoestrogen with antioxidant properties, but its protective effects on osteoblasts under OS and the signaling pathways associated with these effects remain incompletely understood. Here, we evaluated the effects of FMN in ovariectomized (OVX) mice and in a tert-butyl hydroperoxide (t-BOOH)-induced oxidative injury model in MC3T3-E1 cells. Bone microarchitecture, histology, serum OS markers, cell viability, reactive oxygen species (ROS) production, apoptosis, mitochondrial morphology, and osteogenic differentiation were assessed. Network pharmacology, molecular docking, molecular dynamics simulations, RNA sequencing, Western blotting, and pharmacological pathway-modulation experiments were used to investigate signaling changes associated with FMN treatment. FMN attenuated trabecular bone deterioration and systemic oxidative imbalance in OVX mice. In MC3T3-E1 cells, FMN improved cell viability, reduced ROS accumulation and apoptosis, alleviated mitochondrial damage, and preserved osteogenic differentiation following t-BOOH exposure. FMN treatment was accompanied by reduced phosphorylation of extracellular signal-regulated kinase 1/2 (ERK1/2), p38, and c-Jun N-terminal kinase (JNK), together with reduced expression of activator protein 1 (AP-1)-related proteins, including c-Fos, c-Jun, and phosphorylated c-Jun. Pharmacological reactivation of p38/JNK signaling with anisomycin attenuated several antioxidant, anti-apoptotic, and pro-osteogenic effects associated with FMN treatment, whereas nuclear factor erythroid 2-related factor 2 (NRF2) inhibition partially diminished FMN-mediated protection. Collectively, these findings support an association between the protective effects of FMN and modulation of mitogen-activated protein kinase (MAPK)/AP-1 and NRF2-associated signaling.

## 1. Introduction

Osteoporosis (OP) is a prevalent metabolic skeletal disorder characterized by loss of bone mass and disruption of bone microarchitecture, which consequently increases skeletal fragility and susceptibility to fractures [1]. Postmenopausal osteoporosis (PMOP), the most prevalent form of primary OP, is primarily driven by estrogen deficiency. Estrogen withdrawal disrupts bone remodeling by increasing osteoclast-mediated bone resorption and impairing bone formation, ultimately resulting in progressive bone loss [2]. Globally, approximately one-third of women and one-fifth of men aged over 50 years are estimated to experience osteoporotic fractures, with hip fractures posing a particularly serious clinical burden because of their substantial morbidity and mortality [3]. Thus, effective strategies to preserve bone mass and restore the balance between bone resorption and formation are needed to prevent disease progression and its associated fractures.

Current anti-osteoporotic therapies mainly comprise anti-resorptive and bone-forming agents. Anti-resorptive drugs, including bisphosphonates and denosumab, are widely used to suppress osteoclast-mediated bone resorption and reduce fracture risk. Nevertheless, these therapeutic approaches remain limited in their ability to recover compromised bone formation or reverse pre-existing damage to bone microarchitecture. Moreover, prolonged treatment has been linked to uncommon but clinically significant adverse events, such as osteonecrosis of the jaw and atypical femoral fractures [4]. Thus, strategies that preserve osteoblast survival and enhance osteogenic function may be particularly important for restoring bone formation and maintaining skeletal integrity in PMOP.

Oxidative stress (OS) is increasingly considered a key factor involved in the onset and progression of OP [5,6]. Estrogen deficiency promotes the excessive accumulation of reactive oxygen species (ROS), which can disrupt mitochondrial homeostasis, activate apoptotic signaling and impair osteoblast differentiation [7,8]. Under conditions of OS, osteoblasts show decreased expression of osteogenic markers together with compromised mineralization, which further disturbs the balance between bone resorption and formation [9,10]. Thus, mitigating OS-induced osteoblast apoptosis and dysfunction may represent a promising therapeutic strategy for PMOP. Mitogen-activated protein kinases (MAPKs) are critical intracellular signaling molecules that mediate cellular responses to stress, inflammation and oxidative injury [11]. Within the MAPK family, p38 and c-Jun N-terminal kinase (JNK) are widely recognized as kinases activated in response to cellular stress, while extracellular signal-regulated kinase (ERK) is critically involved in regulating cell survival, differentiation, and apoptosis [12,13]. OS is capable of activating MAPK signaling, which subsequently modulates downstream transcription factors such as activator protein 1 (AP-1). AP-1 consists predominantly of proteins from the Jun and Fos families, including c-Jun and c-Fos, and participates in the transcriptional regulation of genes associated with inflammation, apoptosis, and cellular stress responses [14]. Activation of p38 and JNK can promote AP-1 activity and c-Jun phosphorylation, thereby contributing to OS-related cellular injury [15,16,17,18]. Collectively, these findings suggest that the MAPK/AP-1 axis may represent an important signaling pathway linking OS to osteoblast dysfunction.

Formononetin (FMN) is a 7-hydroxyisoflavone primarily derived from red clover (*Trifolium pratense*) and *Astragalus membranaceus* [19]. As a phytoestrogen, FMN has been reported to exhibit anti-inflammatory, antioxidant, anti-apoptotic and estrogenic activities [20,21,22,23,24,25,26,27,28]. Previous studies have suggested that FMN may exert anti-osteoporotic effects by modulating osteoblast and osteoclast function, attenuating bone loss in ovariectomized (OVX) animals, inhibiting adipogenesis and promoting bone regeneration [29,30,31,32]. However, whether FMN protects osteoblasts against OS-induced apoptosis and osteogenic dysfunction in the context of PMOP, and whether these effects involve the MAPK/AP-1 pathway remain unclear.

In the present study, we evaluated the effects of FMN using an OVX mouse model and further examined its protective role against tert-butyl hydroperoxide (t-BOOH)-induced oxidative injury in MC3T3-E1 cells. By integrating in vivo experiments, cellular functional assays, network pharmacology, molecular docking, molecular dynamics simulations, transcriptomic analysis, and pharmacological pathway-modulation experiments, we investigated whether the effects of FMN are associated with alterations in MAPK/AP-1 and nuclear factor erythroid 2-related factor 2 (NRF2)-related signaling during OS, apoptosis, and osteogenic dysfunction.

## 2. Materials and Methods

### 2.1. Reagents and Antibodies

FMN (purity, 98.99%) was purchased from MedChemExpress (HY-N0183, Shanghai, China). Estradiol valerate (E_2_) tablets were obtained from Bayer (J20130009, Berlin, Germany). Sodium carboxymethyl cellulose (CMC-Na) was purchased from Macklin (C804625, Shanghai, China).

Primary antibodies targeting B-cell lymphoma 2 (BCL-2; #3498), BCL-2-associated X protein (BAX; #2772), caspase-3/cleaved caspase-3 (#9662), ERK1/2 (#4695), phosphorylated ERK1/2 (p-ERK1/2; #5726), p38 (#9212), phosphorylated p38 (p-p38; #8690), JNK (#9252), and β-Tubulin (#2146) were obtained from Cell Signaling Technology (Danvers, MA, USA) and applied at a 1:1000 dilution. Antibodies against superoxide dismutase 1 (SOD1; JF1005), heme oxygenase 1 (HO-1; PSH02-76), catalase (CAT; JM11-12), glutathione peroxidase 1 (GPX1; JJ092-07), runt-related transcription factor 2 (RUNX2; SD208-0), Osterix (OSX; PSH07-29), osteocalcin (OCN; ER1919-20), c-Jun (ET1608-3), phosphorylated c-Jun (p-c-Jun; ET1608-4), and glyceraldehyde-3-phosphate dehydrogenase (GAPDH; SA30-01) were purchased from HUABIO (Zhejiang, China). Except for GAPDH, which was used at a dilution of 1:50,000, all other antibodies from HUABIO were used at 1:1000 for Western blotting. The NRF2 antibody (A21176) was purchased from ABclonal (Wuhan, China) and used at 1:1000. The p-JNK antibody (sc-6254) was purchased from Santa Cruz Biotechnology (Dallas, TX, USA) and used at 1:200. The c-Fos antibody (#AF5354) was purchased from Affinity Biosciences (Liyang, China) and used at 1:1000. The cyanine 3 (Cy3)-conjugated goat anti-rabbit immunoglobulin G (IgG; AS007) and Cy3-conjugated goat anti-mouse IgG (heavy and light chains; AS008) secondary antibodies were purchased from ABclonal (Wuhan, China).

t-BOOH solution (#418064) was purchased from Merck (Darmstadt, Germany). Anisomycin (HY-18982) and ML385 (HY-100523) were purchased from MedChemExpress. The following reagents and assay kits were obtained from Beyotime Biotechnology (Shanghai, China): Malondialdehyde (MDA) Assay Kit (S0131S), Superoxide Dismutase (SOD) Assay Kit (S0101S), ROS Assay Kit (S0033M), bicinchoninic acid (BCA) Protein Concentration Assay Kit (P0012), 5-bromo-4-chloro-3-indolyl phosphate/nitro blue tetrazolium (BCIP/NBT) Alkaline Phosphatase (ALP) Color Development Kit (C3206), TRIzol reagent (R0016), Protease and Phosphatase Inhibitor Cocktail (50×, P1045), terminal deoxynucleotidyl transferase dUTP nick-end labeling (TUNEL) Apoptosis Detection Kit (C1089), and 4′,6-diamidino-2-phenylindole (DAPI) staining solution (P0131). Alizarin Red S (ARS) Solution was obtained from OriCell (ALIR10001, Suzhou, China). The Apoptosis Detection Kit was provided by Elabscience (E-CK-A211, Wuhan, China). Cell Counting Kit-8 (CCK-8) was purchased from GLPBIO (GK10001, Montclair, CA, USA). Dexamethasone (D4902), β-glycerophosphate (G9422), and ascorbic acid (PHR1008) were supplied by Sigma-Aldrich (St. Louis, MO, USA).

### 2.2. Animals and Treatments

All animal procedures were approved by the Animal Ethics Committee of Guangzhou University of Chinese Medicine (Approval No. 20250227073). Eight-week-old female C57BL/6J mice weighing approximately 18–20 g were obtained from the Laboratory Animal Center of Guangzhou University of Chinese Medicine. The mice were housed at 22–24 °C and 50–60% relative humidity under a 12 h light/dark cycle, with free access to standard laboratory rodent diet and water.

Following a 7-day acclimatization period, mice were randomly assigned to undergo bilateral ovariectomy or sham surgery using a random-number table. Surgery was performed under sodium pentobarbital anesthesia (50 mg/kg), supplemented with local infiltration of 0.25% bupivacaine (7 μL) at the incision site. In the Sham group, the ovaries were exposed but not removed. Postoperative analgesia was provided with meloxicam (5 mg/kg, intraperitoneally), and penicillin (40,000 units/mouse/day, intraperitoneally) was administered for postoperative infection prophylaxis. Both treatments were administered once daily for three consecutive days after surgery. Animal welfare was assessed twice daily during the first three postoperative days, daily on postoperative days 4–7, and weekly thereafter. Humane endpoints were established for severe postoperative complications and clinical conditions that compromised the animals’ ability to obtain food or water.

Forty-five days after surgery, OVX mice were further randomized using a random-number table into four groups: OVX, OVX plus low-dose FMN (OVX+L-FMN, 15 mg/kg/day), OVX plus high-dose FMN (OVX+H-FMN, 30 mg/kg/day), and OVX plus E_2_ (OVX+E_2_, 0.104 mg/kg/day). Together with the Sham group, the study comprised five groups, with six mice per group. FMN and E_2_ were separately suspended in 0.5% CMC-Na and administered by daily oral gavage for 45 consecutive days. The Sham and untreated OVX groups received an equivalent volume of 0.5% CMC-Na by oral gavage according to the same administration schedule. At the experimental endpoint, blood was collected under anesthesia, followed by euthanasia with sodium pentobarbital (150 mg/kg, i.p.). No specific inclusion or exclusion criteria were established a priori, and no animal deaths, withdrawals, or exclusions occurred during the experimental period.

### 2.3. Micro-Computed Tomography (Micro-CT)

The collected mouse femurs were fixed in 4% paraformaldehyde for 24 h and scanned using an NMC-200 micro-CT scanner (NEMO, PINGSENG Healthcare Inc., Kunshan, China). The system has a nominal spatial resolution of approximately 7.5 μm at 10% modulation transfer function (MTF). Scanning was performed at 90 kV and 50 μA. Projection data were reconstructed using Avatar software (version 1.7) with the Feldkamp–Davis–Kress (FDK) algorithm at a reconstruction matrix of 1024 × 1024 pixels and a nominal reconstructed voxel size of 2 μm. The reconstructed voxel size represents the digital sampling interval of the reconstructed volume and should not be interpreted as the effective spatial resolution of the imaging system. For trabecular bone analysis, the region of interest (ROI) was defined in the distal femoral metaphysis, beginning 0.5 mm proximal to the distal femoral growth plate and extending 1.0 mm proximally, with cortical bone excluded. Image processing, segmentation, and morphometric analysis were also performed using Avatar software. During segmentation, a standardized display window was applied, with a window level of 2000 and a window width of 1800, corresponding to a displayed gray-level range of 1100–2900. The same reconstruction, image-processing, and segmentation settings were applied to all specimens. Trabecular bone morphometric parameters, including bone volume fraction (BV/TV), trabecular thickness (Tb.Th), structure model index (SMI), trabecular number (Tb.N), and trabecular separation (Tb.Sp), were subsequently quantified.

### 2.4. Hematoxylin and Eosin (H&E) Staining

Bone samples were decalcified in 10% EDTA for 4 weeks and subsequently dehydrated through a graded ethanol series, whereas major organs were fixed for preliminary histological evaluation of safety. All processed specimens were then embedded in paraffin and sectioned at a thickness of 4.5 μm. The sections were deparaffinized, rehydrated, and stained with H&E according to routine histological procedures. Images were acquired using an optical microscope. For quantitative analysis of distal femoral sections, three microscopic fields were randomly selected per section and analyzed using ImageJ (version 1.52v). The trabecular bone area was normalized to the corresponding tissue area.

### 2.5. Serum Biochemical Analysis

Under anesthesia, blood samples were collected from the retro-orbital venous plexus and maintained at room temperature for 30 min to allow clot formation. Serum was recovered by centrifugation at 1500× *g* and 4 °C for 10 min and stored at −80 °C until analysis. SOD activity and MDA concentration were determined using commercial assay kits according to the manufacturer’s instructions. Each serum sample was measured in technical triplicate, and the mean of the three measurements was used for subsequent statistical analysis.

### 2.6. Integrated Network Pharmacology and Computational Simulation Analysis

Potential targets of FMN were retrieved from the TCMSP (v2.3), HERB, SwissTargetPrediction, and ITCM databases, while osteoporosis-related targets were collected from GeneCards, DisGeNET, and OMIM using “osteoporosis” as the search term. All databases were accessed on 9 December 2025. For TCMSP, the screening criteria were oral bioavailability (OB) ≥ 30% and drug-likeness (DL) ≥ 0.18. TCMSP-derived targets were mapped to standardized human gene symbols using UniProt, and unmapped entries and duplicates were removed. Overlapping targets between FMN and osteoporosis were identified using Venny (version 2.1) and subsequently submitted to STRING for protein–protein interaction (PPI) network construction. STRING analysis was performed using the multiple-proteins mode, with the species restricted to Homo sapiens and the minimum required interaction score set to 0.900. Gene Ontology (GO) and Kyoto Encyclopedia of Genes and Genomes (KEGG) enrichment analyses were performed using the bioinformatics online platform (https://www.bioinformatics.com.cn/; accessed on 9 December 2025), based on the hypergeometric test implemented in the clusterProfiler package (version 4.18.1). The enrichment *p* values were not adjusted for multiple testing, and a nominal *p* value < 0.05 was used as an exploratory threshold. Accordingly, these enrichment analyses were considered exploratory and were used for pathway prioritization and hypothesis generation rather than confirmatory statistical inference.

For molecular docking, the three-dimensional structure of FMN was obtained from PubChem (https://pubchem.ncbi.nlm.nih.gov/; accessed on 9 December 2025) and converted from structure-data file (SDF) format to Protein Data Bank (PDB) format using Open Babel 3.1.1. The crystal structures of MAPK1, MAPK8, and MAPK14 (PDB IDs: 8AOJ, 4QTD, and 3LFF, respectively) were retrieved from the RCSB Protein Data Bank (https://www.rcsb.org/; accessed on 9 December 2025). Water molecules and native ligands were removed using PyMOL 3.1.0, followed by hydrogen addition and Gasteiger charge assignment using AutoDock Tools 1.5.7. Molecular docking was performed using AutoDock Vina 1.2.6. The grid center coordinates and box dimensions for each protein are provided in Supplementary Table S3. The docking poses with the lowest predicted binding energies were selected for visualization and subsequent molecular dynamics simulations.

Molecular dynamics simulations of the FMN–MAPK complexes were performed for 100 ns using GROMACS 2022.2 [33]. The Amber99SB-ILDN force field was applied to the proteins [34], whereas ligand parameters were generated using sobtop 1.0 (dev3.1) based on the General Amber Force Field 2 (GAFF2) and restrained electrostatic potential (RESP)-derived atomic charges. Each complex was solvated in a cubic three-point transferable intermolecular potential (TIP3P) water box, with a minimum distance of 1.0 nm between the protein and the box boundary. Na^+^ and Cl^−^ ions were added to neutralize the systems and establish a 0.15 M NaCl environment. Long-range electrostatic interactions were calculated using the particle mesh Ewald method, with cutoff distances of 1.2 nm for short-range electrostatic and van der Waals interactions. Covalent bonds were constrained using the Linear Constraint Solver (LINCS) algorithm, and an integration time step of 2 fs was applied. Following energy minimization using the steepest descent and conjugate gradient methods, each system underwent 10 ps of canonical ensemble (NVT) equilibration and 10 ps of isothermal–isobaric ensemble (NPT) equilibration with positional restraints applied to the proteins and ligands. Temperature and pressure were maintained at 300 K and 1 bar using the V-rescale thermostat and Parrinello–Rahman barostat, respectively. Unrestrained production simulations were subsequently conducted. Trajectories were analyzed for root mean square deviation (RMSD), root mean square fluctuation, radius of gyration (Rg), solvent-accessible surface area, and protein–ligand hydrogen bonds using GROMACS tools (version 2022.2). Free energy landscapes were generated using the GROMACS gmx sham utility, with RMSD and Rg as the reaction coordinates. Molecular mechanics/Poisson–Boltzmann surface area (MM-PBSA) binding-energy estimates and per-residue energy decomposition were calculated using 11 snapshots extracted from the final 10 ns (90–100 ns) of each trajectory at 1 ns intervals.

### 2.7. Cell Culture and Treatments

MC3T3-E1 subclone 14 cells (GNM15) were obtained from the Cell Bank of the Chinese Academy of Sciences (Shanghai, China). According to the quality-control report provided by the cell bank, the cells tested negative for Mycoplasma contamination. Cells between passages 5 and 15 were used for all experiments. Cells were cultured in α-minimum essential medium (α-MEM) supplemented with 10% fetal bovine serum and 1% penicillin–streptomycin at 37 °C in a humidified atmosphere containing 5% CO_2_. All cell culture reagents were obtained from Gibco (Grand Island, NY, USA), and the culture medium was replaced every other day. FMN and ML385 were dissolved in dimethyl sulfoxide (DMSO) and diluted with culture medium to the required concentrations. The final DMSO concentration did not exceed 0.1% (*v*/*v*), and vehicle controls received an equivalent concentration of DMSO.

For OS experiments, cells were pretreated with FMN for 2 h before exposure to 100 μM t-BOOH for 24 h, with FMN maintained during t-BOOH exposure. For experiments involving p38/JNK reactivation, cells were pretreated with FMN for 2 h and subsequently exposed to 0.16 μM anisomycin for 1 h. The anisomycin-containing medium was then removed, and cells were treated with FMN and 100 μM t-BOOH for an additional 24 h. For NRF2 inhibition experiments, cells were pretreated with 5 μM ML385 for 2 h, followed by the addition of FMN and 100 μM t-BOOH. ML385 was maintained in the culture medium throughout the subsequent 24 h treatment period.

### 2.8. Cell Viability Assay

MC3T3-E1 cells were seeded in 96-well plates at 5 × 10^3^ cells/well and subjected to the indicated treatments. Subsequently, 10 μL of CCK-8 reagent was added to each well containing 100 μL of culture medium, followed by incubation at 37 °C for 2 h. Absorbance was measured at 450 nm using a microplate reader.

### 2.9. Apoptosis Assay

After the indicated treatments, cells were collected and stained with Annexin V–fluorescein isothiocyanate (FITC) and propidium iodide (PI) for 15 min at room temperature in the dark. The stained cells were analyzed using a BD LSRFortessa flow cytometer (BD Biosciences, San Jose, CA, USA), and the data were processed with FlowJo software (version 10.8). Viable, early apoptotic, and late apoptotic or necrotic cells were identified according to Annexin V-FITC/PI staining, and the total apoptosis rate was calculated as the sum of early and late apoptotic cells.

### 2.10. ROS Measurement

MC3T3-E1 cells were incubated with 10 μM DCFH-DA at 37 °C for 20 min in the dark. Fluorescence images were acquired using a BioTek Cytation C10 imaging system (Agilent Technologies, Winooski, VT, USA) under consistent imaging conditions. ROS-associated fluorescence was quantified using ImageJ. Mean fluorescence intensity was measured within cellular ROIs after subtraction of the background signal obtained from cell-free regions of the corresponding images. The resulting values were normalized to the control group and expressed as relative fluorescence intensity.

### 2.11. Transmission Electron Microscopy (TEM)

After the indicated treatments, MC3T3-E1 cells were fixed with 2.5% glutaraldehyde, post-fixed with 1% osmium tetroxide, dehydrated, and embedded in Epon 812 resin. Ultrathin sections were prepared, stained with uranyl acetate and lead citrate, and examined using a transmission electron microscope (FEI TECNAI G2 20 TWIN, FEI, Eindhoven, The Netherlands) to assess cellular ultrastructure, with particular emphasis on mitochondrial morphology.

### 2.12. Osteogenic Differentiation

MC3T3-E1 cells were seeded in 12-well plates at a density of 4 × 10^4^ cells/well. Following a 2 h pretreatment with FMN, cells were exposed to 100 μM t-BOOH for 24 h in the continued presence of FMN. For experiments involving anisomycin, cells were additionally treated with 0.16 μM anisomycin for 1 h following FMN pretreatment, after which anisomycin was removed before t-BOOH exposure.

Following the initial treatments, cells were cultured in osteogenic induction medium containing 10 nM dexamethasone, 10 mM β-glycerophosphate, and 50 μg/mL ascorbic acid. FMN treatment was maintained at the corresponding concentrations throughout osteogenic induction, whereas t-BOOH and anisomycin were not maintained during this period. ALP staining was performed after 7 days using a BCIP/NBT Alkaline Phosphatase Color Development Kit, and matrix mineralization was evaluated after 21 days using ARS staining. Images were acquired using a BioTek Cytation C10 imaging system. ALP and ARS staining intensities were quantified using ImageJ after background subtraction and normalized to the corresponding reference group within each independent experiment.

### 2.13. Transcriptome Analysis

Total RNA was extracted from MC3T3-E1 cells in the t-BOOH and t-BOOH+FMN groups using TRIzol reagent, with three independent biological replicates per group. RNA quality assessment, library preparation, and RNA sequencing (RNA-seq) were performed by Biomarker Technologies Co., Ltd. (Beijing, China). Strand-specific mRNA libraries were sequenced on an Illumina NovaSeq 6000 platform (San Diego, CA, USA) using paired-end 150 bp reads. Following quality control, clean reads were aligned to the mouse reference genome (GRCm38, release 95) using HISAT2 (version 2.2.1). StringTie (version 2.2.1) was used for transcript assembly and gene expression quantification, with expression levels reported as fragments per kilobase of transcript per million mapped fragments (FPKM). Differential expression analysis was performed on gene-level count data using edgeR (version 4.8.0), with *p* values adjusted for multiple testing using the Benjamini–Hochberg false discovery rate (FDR) method. Genes with an FDR < 0.05 and an absolute log_2_ fold change ≥1.5 were identified as differentially expressed genes (DEGs). GO and KEGG enrichment analyses were subsequently performed on the DEGs to explore associated biological functions and signaling pathways. The enrichment *p* values were not adjusted for multiple testing, and a nominal *p* value < 0.05 was used as an exploratory threshold.

### 2.14. TUNEL Staining and Immunofluorescence Analysis

Paraffin-embedded distal femoral sections were deparaffinized, rehydrated, and subjected to antigen retrieval. For immunofluorescence staining, sections were incubated with the appropriate primary antibodies overnight at 4 °C, followed by species-matched Cy3-conjugated secondary antibodies. Specifically, the Cy3-conjugated Goat anti-Mouse IgG was used for p-JNK staining, whereas the Cy3-conjugated Goat anti-Rabbit IgG was used for staining with rabbit-derived primary antibodies. TUNEL staining was performed using a TUNEL Apoptosis Detection Kit, and nuclei were counterstained with DAPI. Fluorescence images were acquired using a BioTek Cytation C10 imaging system. Background-subtracted mean fluorescence intensity was quantified using ImageJ within the selected ROIs and normalized to the Sham group.

### 2.15. Western Blotting

Total protein was isolated by lysing cells in RIPA buffer supplemented with PMSF and a protease and phosphatase inhibitor cocktail. Equivalent amounts of protein (20 μg) were resolved on 12% SDS-polyacrylamide gels and subsequently transferred to PVDF membranes. The membranes were blocked with 5% skimmed milk for 60 min at room temperature and then incubated with the corresponding primary antibodies overnight at 4 °C. Following TBST washing, species-specific HRP-conjugated secondary antibodies were applied for 1 h at room temperature. Protein bands were subsequently detected using an enhanced chemiluminescence (ECL) system, and band intensities were quantified with ImageJ software. For densitometric analysis, target proteins were normalized to their corresponding loading controls from matched experimental samples, except for p-ERK1/2, p-p38, and p-JNK, which were normalized to their respective total protein levels. When different target proteins were analyzed on separate blots, their corresponding loading controls were used for normalization.

### 2.16. Statistical Analysis

All statistical analyses were performed using GraphPad Prism software (version 10.0). Quantitative data are presented as the mean ± standard deviation (SD). The normality of the data distribution was assessed using the Shapiro–Wilk test, and homogeneity of variance was evaluated using the Brown–Forsythe test before parametric analyses. For data satisfying the assumptions of normality and homogeneity of variance, comparisons between two independent groups were performed using an unpaired two-tailed Student’s *t*-test, whereas comparisons among three or more groups were performed using one-way analysis of variance (ANOVA) followed by Tukey’s multiple-comparisons test. When the assumption of homogeneity of variance was not satisfied but the data were approximately normally distributed, Welch’s *t*-test was used for comparisons between two groups, and Welch’s ANOVA followed by Dunnett’s T3 multiple-comparisons test was used for comparisons among three or more groups. For data showing substantial deviation from normality, the Mann–Whitney U test or Kruskal–Wallis test followed by Dunn’s multiple-comparisons test was applied, as appropriate. All statistical tests were two-sided, and a *p* value < 0.05 was considered statistically significant. Statistical significance was indicated as follows: ns, not significant; * *p* < 0.05, ** *p* < 0.01, *** *p* < 0.001, and **** *p* < 0.0001. No a priori sample-size calculation was performed, and no single primary outcome was prespecified. Accordingly, the present study was exploratory in nature. For animal experiments, each mouse was considered an independent biological replicate. Micro-CT analysis included six mice per group. For analyses requiring three biological replicates, three mice were randomly selected from each group using a random-number table before outcome assessment. Measurements obtained from multiple microscopic fields or technical replicates of the same biological sample were averaged to generate a single value per animal for statistical analysis. The exact sample size for each analysis is indicated in the corresponding figure legend. For cell-based functional assays and Western blotting, biological replicates represented independent experiments in which cells were cultured and treated separately. Technical replicate measurements obtained within the same experiment were averaged before statistical analysis. Unless otherwise indicated, *n* = 3 represents three independent biological experiments. Outcome assessment and data analysis for both animal and cell experiments were performed under blinded conditions. Investigators were unaware of group allocation during outcome evaluation, quantification, and statistical analysis.

## 3. Results

### 3.1. FMN Attenuates Bone Loss and Ameliorates OS in OVX Mice

**Figure 1:**
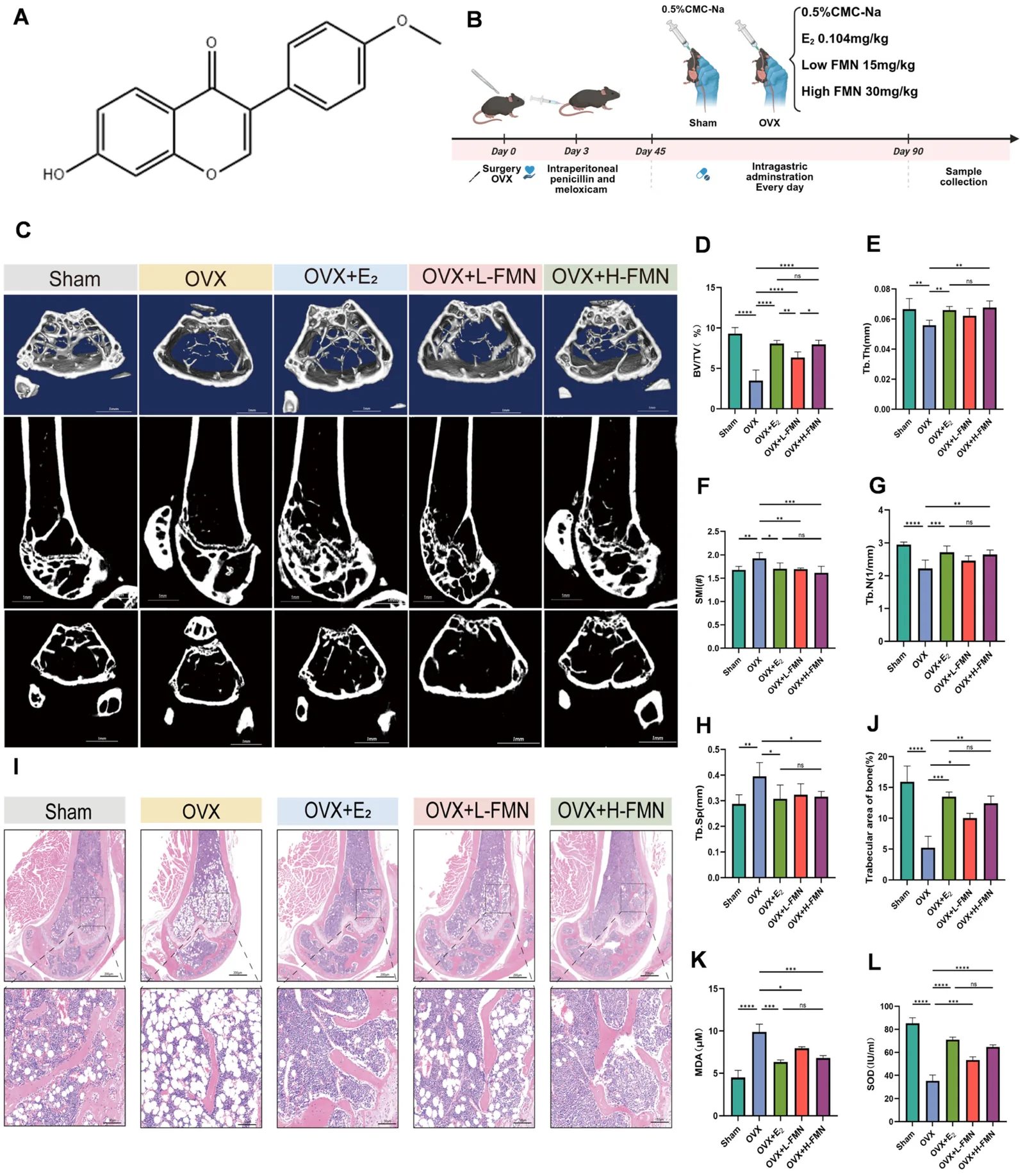
FMN attenuates OVX-induced trabecular bone loss and systemic oxidative stress in mice. (**A**) Chemical structure of FMN. (**B**) Schematic diagram of the animal experimental timeline. (**C**) Representative micro-CT images of the distal femur. Top, 3D reconstruction; middle, sagittal view; bottom, transverse view (*n* = 6, scale bar = 1 mm). (**D**–**H**) Quantitative analysis of trabecular bone microarchitectural parameters, including BV/TV (**D**), Tb.Th (**E**), SMI (**F**), Tb.N (**G**), and Tb.Sp (**H**) (*n* = 6). (**I**) Representative H&E-stained sections of the distal femur. Scale bars indicate 200 μm in the upper panels and 50 μm in the lower panels (*n* = 3). (**J**) Quantitative analysis of trabecular bone area per unit tissue area in the distal femur (*n* = 3). (**K**) Serum malondialdehyde (MDA) levels (*n* = 3). (**L**) Serum superoxide dismutase (SOD) activity (*n* = 3). OVX, ovariectomy; E_2_, estradiol valerate; L-FMN, low-dose FMN; H-FMN, high-dose FMN. Data are presented as mean ± SD; *n* represents the number of individual mice. Statistical comparisons were performed using one-way ANOVA followed by Tukey’s multiple-comparisons test. ns, not significant; * *p* < 0.05, ** *p* < 0.01, *** *p* < 0.001, and **** *p* < 0.0001.

To evaluate the effects of FMN on PMOP, an OVX mouse model was established. The chemical structure of FMN is shown in Figure 1A. Beginning 45 days after surgery, OVX mice received daily intragastric administration of low-dose FMN (15 mg/kg), high-dose FMN (30 mg/kg), or the positive control E_2_ (0.104 mg/kg) until sample collection (Figure 1B). To provide additional morphological evidence supporting the establishment of the OVX model, gross uterine morphology was examined. Representative photographs revealed visibly smaller uteri in OVX mice than in Sham mice, consistent with OVX-associated uterine atrophy (Supplementary Figure S1D). The effects of FMN on OVX-induced bone loss and systemic OS were assessed using micro-CT, femoral histological staining and serum OS marker assays.

Micro-CT analysis of the distal femur showed marked deterioration of trabecular bone microarchitecture in OVX mice compared with Sham mice (Figure 1C). Quantitative analysis revealed that OVX significantly reduced BV/TV, Tb.Th, and Tb.N, while increasing Tb.Sp and SMI (Figure 1D–H). Treatment with E_2_ or FMN partially restored these microarchitectural parameters. Notably, high-dose FMN produced a more pronounced protective effect than low-dose FMN, as indicated by higher BV/TV and Tb.N and lower Tb.Sp and SMI. These findings indicate that FMN attenuates OVX-induced deterioration of trabecular bone microarchitecture, with a greater effect observed at the higher dose.

Consistent with the micro-CT findings, H&E staining of femoral sections showed abundant, continuous trabecular structures in the Sham group, whereas OVX mice exhibited sparse and disrupted trabeculae with enlarged marrow spaces (Figure 1I). Treatment with E_2_ or FMN attenuated these histological abnormalities, with greater preservation of trabecular architecture observed in the OVX+H-FMN group than in the OVX+L-FMN group. Quantitative analysis further showed that OVX markedly reduced the trabecular area fraction, whereas E_2_ and FMN treatment significantly increased trabecular bone area (Figure 1J).

To determine whether FMN alleviated OVX-related OS, serum MDA levels and SOD activity were evaluated. Relative to the Sham group, OVX resulted in a significant elevation of serum MDA levels accompanied by a reduction in SOD activity, indicating enhanced systemic OS following estrogen deficiency (Figure 1K,L). FMN treatment attenuated these changes, with high-dose FMN significantly reducing MDA levels and increasing SOD activity. The magnitude and direction of these changes in the high-dose FMN group were descriptively similar to those observed in the E_2_-treated group.

In addition, RUNX2 immunofluorescence staining was performed to assess osteoblast-associated activity in the distal femur. OVX markedly reduced RUNX2 fluorescence intensity compared with the Sham group, whereas FMN treatment partially restored RUNX2 expression, particularly at the higher dose (Supplementary Figure S1A,B). Histological examination of the heart, liver, spleen, lung, and kidney revealed no obvious histological abnormalities associated with FMN administration at the tested doses under the present experimental conditions (Supplementary Figure S1C).

Collectively, these findings suggest that FMN attenuates OVX-associated trabecular bone deterioration, improves circulating OS markers, and partially preserves RUNX2-associated fluorescence in bone tissue.

### 3.2. Network Pharmacology, Molecular Docking, and Molecular Dynamics Simulations Implicate the MAPK Signaling Pathway in the Effects of FMN Against OP

**Figure 2:**
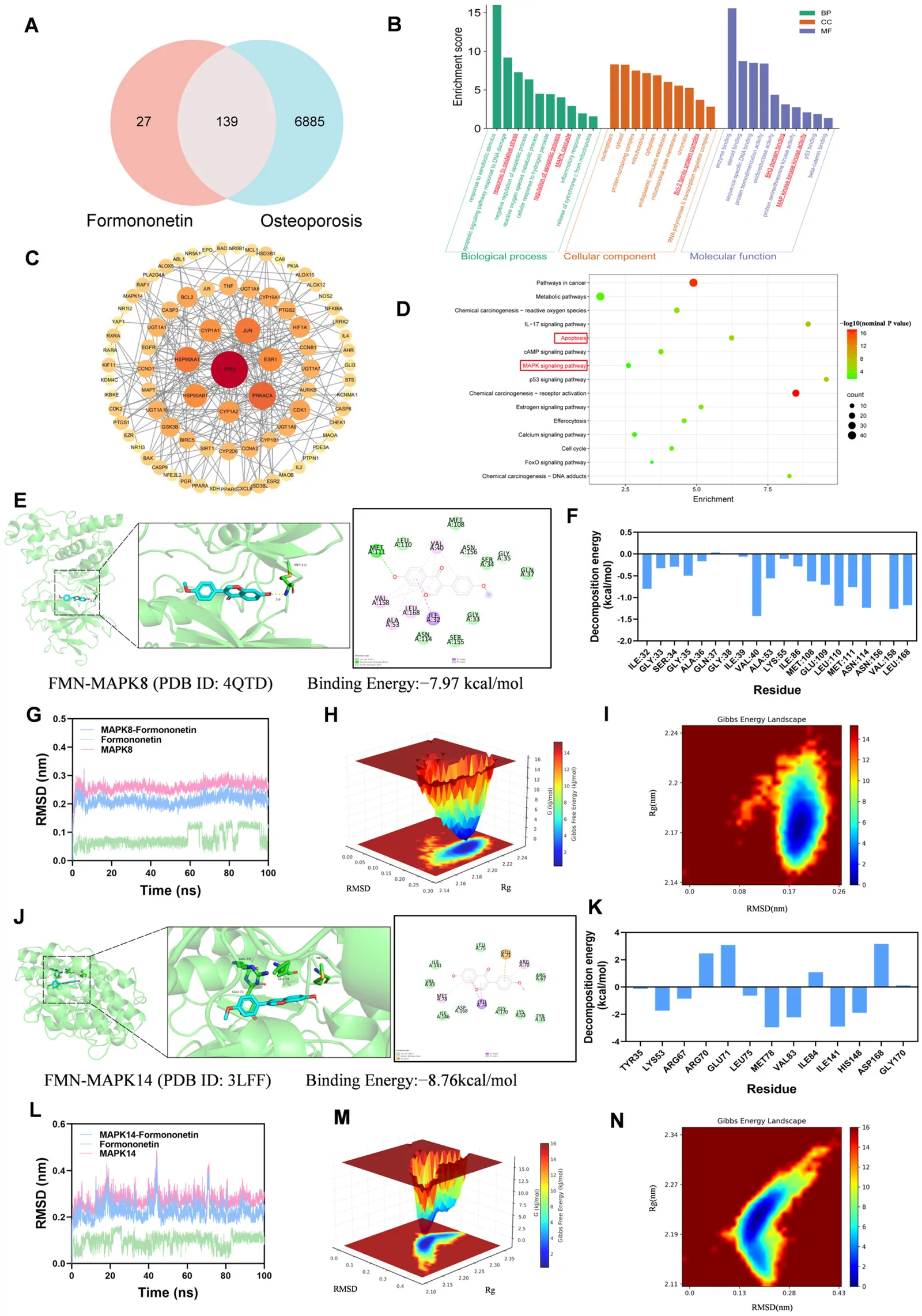
Network pharmacology, molecular docking, and molecular dynamics analyses highlight MAPK signaling as a candidate pathway associated with FMN. (**A**) Venn diagram of FMN-related targets and osteoporosis-related targets. (**B**) GO enrichment analysis of the overlapping targets. (**C**) PPI network of the overlapping targets. (**D**) KEGG pathway enrichment analysis of the overlapping targets. *p* values for the GO and KEGG enrichment analyses were not adjusted for multiple testing. (**E**) Molecular docking analysis of FMN with MAPK8, including the overall docking conformation, enlarged binding pocket, and two-dimensional interaction diagram. (**F**) Residue energy decomposition analysis of the FMN–MAPK8 complex. (**G**) RMSD plot of the FMN–MAPK8 complex during 100 ns molecular dynamics simulation. (**H**,**I**) Gibbs free energy landscape of the FMN–MAPK8 complex. (**J**) Molecular docking analysis of FMN with MAPK14, including the overall docking conformation, enlarged binding pocket, and two-dimensional interaction diagram. (**K**) Residue energy decomposition analysis of the FMN–MAPK14 complex. (**L**) RMSD plot of the FMN–MAPK14 complex during 100 ns molecular dynamics simulation. (**M**,**N**) Gibbs free energy landscape of the FMN–MAPK14 complex.

To investigate the potential molecular mechanisms underlying the effects of FMN against OP, network pharmacology analysis was first performed. A total of 166 potential targets of FMN were obtained from TCMSP, HERB, SwissTargetPrediction, and ITCM (Supplementary Table S1), while 7024 OP-associated targets were collected from OMIM, DisGeNET, and GeneCards. Intersection analysis revealed 139 shared targets, which were considered potential therapeutic targets of FMN for OP (Figure 2A; Supplementary Table S2). To explore the interactions among these targets, a PPI network was subsequently established using STRING (Figure 2C).

GO enrichment analysis showed that the overlapping targets were primarily involved in biological processes related to OS, apoptosis, ROS metabolism and the MAPK cascade. Cellular component analysis highlighted enrichment in the nucleoplasm, cytoplasmic region and BCL-2 family protein complexes, whereas molecular function analysis showed enrichment in enzyme binding, oxidoreductase activity, BCL-2 homology 3 (BH3) domain binding and MAP kinase activity (Figure 2B). KEGG pathway analysis further highlighted several enriched signaling pathways, including chemical carcinogenesis–ROS, interleukin-17 (IL-17) signaling, apoptosis and, notably, the MAPK signaling pathway (Figure 2D). Based on these enrichment results, the MAPK signaling pathway was prioritized for subsequent computational analyses and experimental evaluation.

To further assess the network pharmacology predictions, molecular docking was performed between FMN and three representative MAPK family proteins: MAPK1 (ERK2), MAPK8 (JNK1), and MAPK14 (p38α). FMN showed favorable predicted binding affinities for MAPK1, MAPK8, and MAPK14, with binding energies of −6.95, −7.97 and −8.76 kcal/mol, respectively, suggesting favorable interactions between FMN and the predicted binding sites of these kinases (Supplementary Figure S2A; Figure 2E,J).

The dynamic stability of the docking-predicted protein–ligand complexes was further evaluated using 100 ns molecular dynamics simulations. RMSD analysis showed that the protein–ligand complexes remained relatively stable throughout the simulations, with the systems reaching dynamic equilibrium after the initial equilibration phase (Figure 2G,L; Supplementary Figure S2C). Residue energy decomposition analysis identified several amino acid residues that contributed substantially to the calculated ligand-binding energy, suggesting that these residues may contribute to stabilizing FMN within the predicted binding pockets of MAPK8, MAPK14 and MAPK1 (Figure 2F,K; Supplementary Figure S2B). The Rg values showed only minor fluctuations throughout the simulations, indicating that the three protein–ligand complexes maintained relatively stable and compact conformations without apparent structural expansion or collapse (Supplementary Figure S2F). Furthermore, Gibbs free energy landscape analysis revealed distinct low-energy conformational regions for all three FMN–MAPK complexes, suggesting that the simulated systems sampled relatively favorable conformational states (Figure 2H,I,M,N; Supplementary Figure S2D,E). Together, these computational analyses support a potential role of MAPK signaling in the effects of FMN against OP.

### 3.3. FMN Protects MC3T3-E1 Cells from t-BOOH-Induced Cytotoxicity and Apoptosis

**Figure 3:**
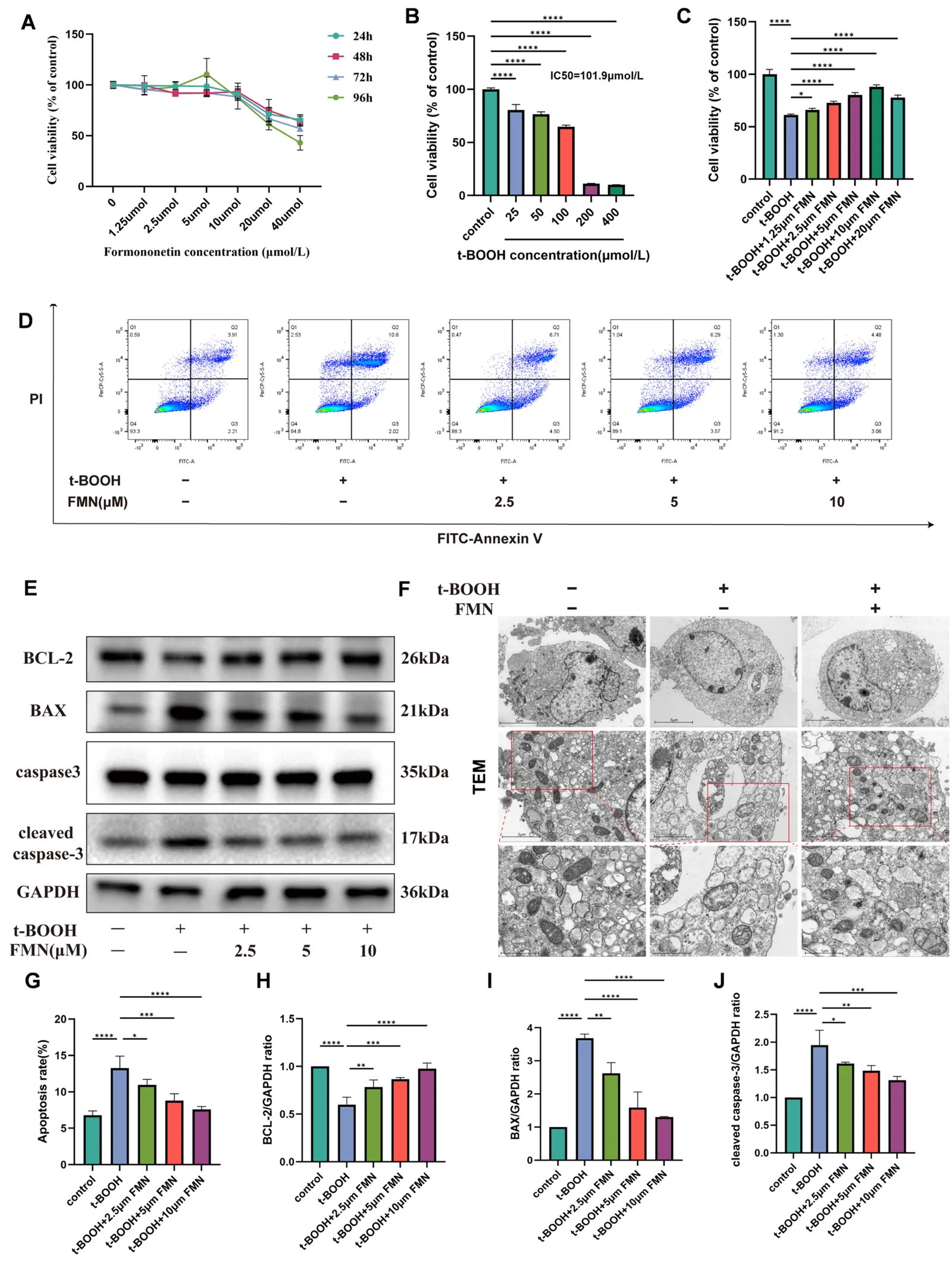
FMN protects MC3T3-E1 cells against t-BOOH-induced cytotoxicity, apoptosis, and mitochondrial damage. (**A**) Cell viability of MC3T3-E1 cells after treatment with FMN at 1.25, 2.5, 5, 10, 20, and 40 μM for 24, 48, 72, and 96 h. (**B**) Cell viability of MC3T3-E1 cells after treatment with t-BOOH at 25, 50, 100, 200, and 400 μM for 24 h. (**C**) Cell viability of t-BOOH-treated MC3T3-E1 cells after intervention with FMN at 1.25, 2.5, 5, 10, and 20 μM. (**D**) Representative Annexin V-FITC/PI apoptosis plots. (**E**) Western blot analysis of BCL-2, BAX, caspase-3, and cleaved caspase-3 using GAPDH as the internal reference. (**F**) Representative TEM images showing cellular and mitochondrial ultrastructure under the indicated treatment conditions. The middle panels show enlarged views of cellular ultrastructure, and the bottom panels show higher-magnification views of the boxed regions. Scale bars: 5 μm (top), 2 μm (middle), and 1 μm (bottom). (**G**) Quantitative analysis of apoptosis rate. (**H**–**J**) Quantitative analysis of BCL-2 (**H**), BAX (**I**), and cleaved caspase-3 (**J**) protein levels. Data are presented as mean ± SD from three independent biological experiments (*n* = 3). Statistical comparisons were performed using one-way ANOVA followed by Tukey’s multiple-comparisons test. ns, not significant; * *p* < 0.05, ** *p* < 0.01, *** *p* < 0.001, and **** *p* < 0.0001.

An in vitro oxidative injury model was established by exposing MC3T3-E1 cells to t-BOOH. The effects of FMN on cell viability were initially assessed over different concentrations and treatment durations. FMN at concentrations of up to 10 μM did not markedly affect cell viability over the tested time range, whereas higher concentrations, particularly with prolonged exposure, tended to reduce cell viability (Figure 3A). t-BOOH reduced the viability of MC3T3-E1 cells in a concentration-dependent manner, yielding a half-maximal inhibitory concentration (IC_50_) of 101.9 μM after 24 h of treatment. Based on these findings, exposure to 100 μM t-BOOH for 24 h was chosen to generate the oxidative injury model (Figure 3B). Compared with t-BOOH treatment alone, FMN significantly increased cell viability across the tested concentrations within the non-cytotoxic range of up to 10 μM, with a greater effect observed at higher concentrations (Figure 3C). These findings indicate that FMN attenuates t-BOOH-induced loss of cell viability under the experimental conditions used.

Flow cytometric analysis showed that t-BOOH markedly increased the proportion of apoptotic cells, whereas FMN treatment reduced the apoptosis rate, with the most pronounced effect observed at 10 μM (Figure 3D,G). In agreement with these observations, Western blot analysis revealed that t-BOOH decreased the expression of the anti-apoptotic protein BCL-2 while elevating cleaved caspase-3 levels. In contrast, FMN treatment reversed these changes by restoring BCL-2 expression and suppressing caspase-3 cleavage after t-BOOH exposure (Figure 3E,H–J). These findings indicate that FMN attenuates t-BOOH-induced apoptosis in MC3T3-E1 cells.

TEM further revealed ultrastructural changes in MC3T3-E1 cells. Control cells exhibited relatively intact cellular morphology, with well-preserved mitochondrial structure and clearly visible cristae. In contrast, t-BOOH-treated cells showed mitochondrial swelling, disrupted cristae and nuclear shrinkage. FMN treatment attenuated these ultrastructural abnormalities, as evidenced by relatively preserved mitochondrial morphology and reduced cellular structural damage (Figure 3F). Collectively, these findings indicate that FMN attenuates t-BOOH-induced cytotoxicity, apoptosis and mitochondrial ultrastructural damage in MC3T3-E1 cells.

### 3.4. FMN Reduces OS and Preserves Osteogenic Differentiation Under t-BOOH-Induced OS

**Figure 4:**
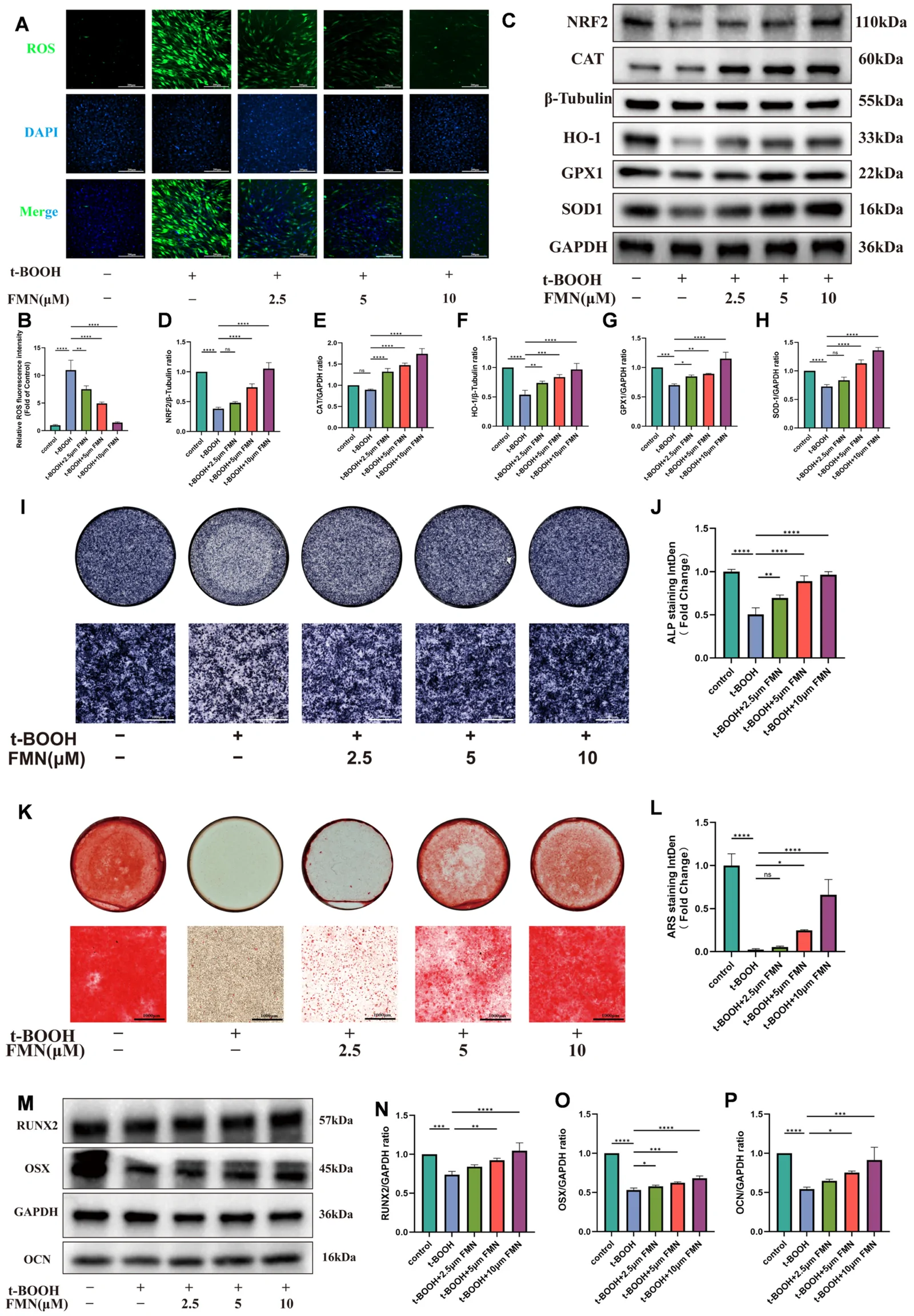
FMN reduces t-BOOH-induced oxidative stress and preserves osteogenic differentiation in MC3T3-E1 cells. (**A**) Representative fluorescence images of intracellular ROS staining. Nuclei were counterstained with DAPI. (**B**) Quantitative analysis of ROS fluorescence intensity. (**C**) Representative Western blots of NRF2, CAT, HO-1, GPX1, and SOD1, each displayed with its corresponding loading control. NRF2 and HO-1 were normalized to β-Tubulin, whereas CAT, GPX1, and SOD1 were normalized to GAPDH. (**D**–**H**) Quantitative analysis of NRF2/β-Tubulin (**D**), CAT/GAPDH (**E**), HO-1/β-Tubulin (**F**), GPX1/GAPDH (**G**), and SOD1/GAPDH (**H**). (**I**,**J**) Representative ALP staining images and quantitative analysis of ALP staining intensity. (**K**,**L**) Representative ARS staining images and quantitative analysis of mineralized nodule formation. (**M**) Western blot analysis of RUNX2, OSX, and OCN using GAPDH as the internal reference. (**N**–**P**) Quantitative analysis of RUNX2 (**N**), OSX (**O**), and OCN (**P**) protein levels. Data are presented as mean ± SD from three independent biological experiments (*n* = 3). Statistical comparisons were performed using one-way ANOVA followed by Tukey’s multiple-comparisons test. ns, not significant; * *p* < 0.05, ** *p* < 0.01, *** *p* < 0.001, and **** *p* < 0.0001.

To further evaluate the effects of FMN under OS conditions, intracellular ROS accumulation and osteogenic differentiation capacity were assessed in t-BOOH-treated MC3T3-E1 cells. ROS staining showed that t-BOOH markedly increased intracellular ROS levels, whereas FMN treatment significantly reduced ROS accumulation, with a greater effect observed at higher concentrations (Figure 4A,B). Consistent with these findings, Western blot analysis showed that t-BOOH impaired the antioxidant defense response, as reflected by reduced expression of NRF2 and its downstream antioxidant proteins, including CAT, HO-1, GPX1, and SOD1. FMN treatment increased the expression of these antioxidant-related proteins, particularly at 5 and 10 μM, suggesting enhanced antioxidant defense in MC3T3-E1 cells under t-BOOH-induced OS (Figure 4C–H). To further assess osteogenic function, t-BOOH markedly reduced ALP staining intensity and mineralized nodule formation, whereas FMN treatment attenuated these inhibitory effects, with greater effects observed at higher concentrations (Figure 4I–L). Consistent with the staining results, Western blot analysis showed that t-BOOH reduced the expression of osteogenic markers, including RUNX2, OSX and OCN, whereas FMN treatment increased the expression of these markers under OS conditions (Figure 4M–P). Collectively, these findings indicate that FMN attenuates t-BOOH-induced OS and preserves osteogenic differentiation in MC3T3-E1 cells, accompanied by enhanced expression of antioxidant defense proteins and osteogenic markers.

### 3.5. Transcriptomic and Protein Analyses Reveal MAPK/AP-1-Associated Signaling Changes Following FMN Treatment

**Figure 5:**
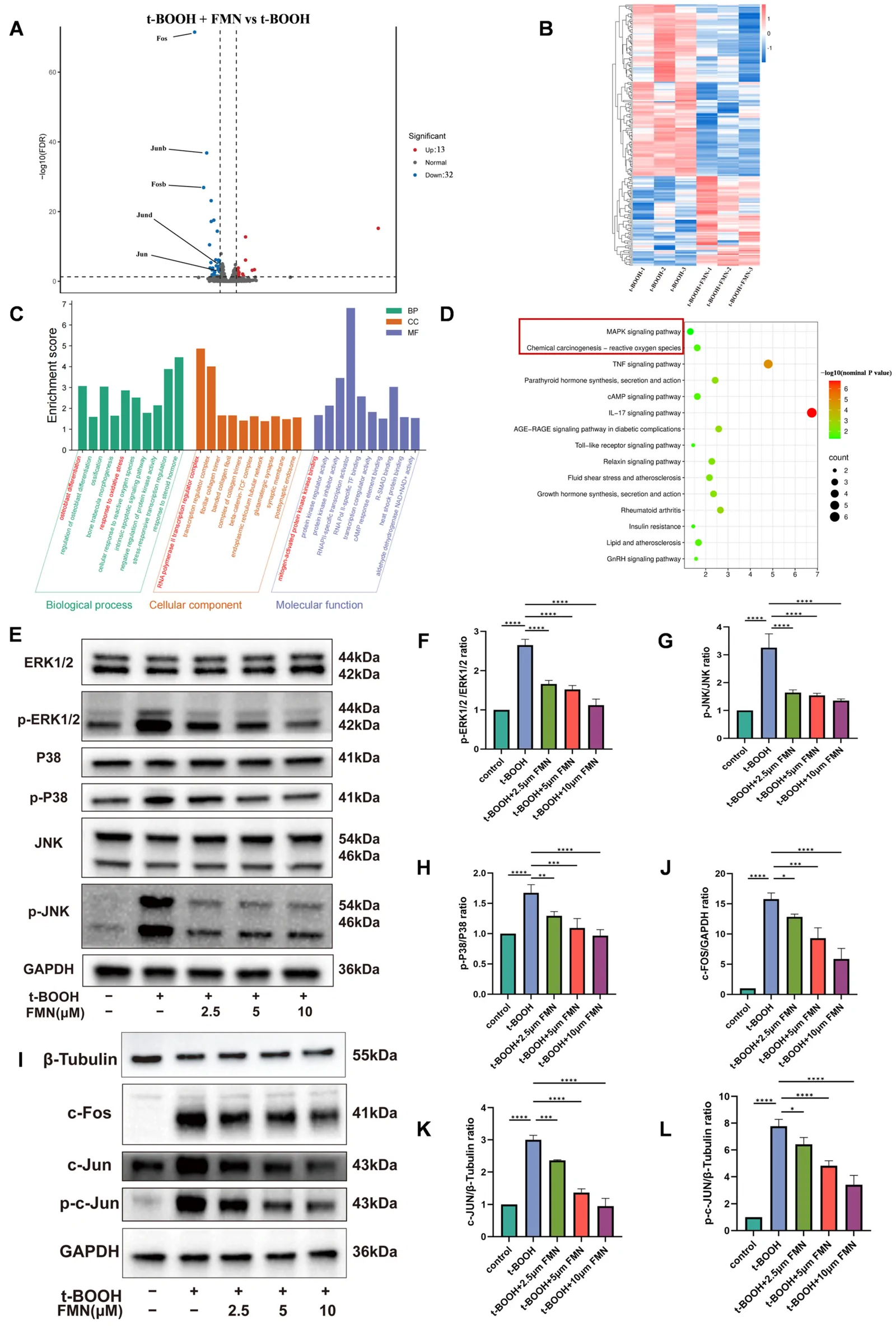
Transcriptomic and protein analyses reveal MAPK/AP-1-associated signaling changes following FMN treatment. (**A**) Volcano plot of differentially expressed genes between the t-BOOH and t-BOOH+FMN groups. AP-1-related genes are labeled. (**B**) Hierarchical clustering heatmap of differentially expressed genes. (**C**) GO enrichment analysis of differentially expressed genes. (**D**) KEGG pathway enrichment analysis of differentially expressed genes. *p* values for the GO and KEGG enrichment analyses were not adjusted for multiple testing. Bubble size represents the number of enriched genes, and color intensity represents −log10(nominal *p* value). (**E**) Western blot analysis of ERK1/2, p-ERK1/2, p38, p-p38, JNK, and p-JNK using GAPDH as the internal reference. (**F**–**H**) Quantitative analysis of p-ERK1/2/ERK1/2 (**F**), p-JNK/JNK (**G**), and p-p38/p38 (**H**). (**I**) Representative Western blots of c-Fos, c-Jun, and p-c-Jun, with selected β-Tubulin and GAPDH bands shown. Each protein was quantified using its corresponding loading control from the original blots. (**J**–**L**) Quantitative analysis of c-Fos/GAPDH (**J**), c-Jun/β-Tubulin (**K**), and p-c-Jun/β-Tubulin (**L**). For RNA-seq, *n* = 3 independent biological replicates per group. For Western blot quantification, data are presented as mean ± SD from three independent biological experiments (*n* = 3). Statistical comparisons were performed using one-way ANOVA followed by Tukey’s multiple-comparisons test. ns, not significant; * *p* < 0.05, ** *p* < 0.01, *** *p* < 0.001, and **** *p* < 0.0001.

To characterize the transcriptional changes associated with the protective effects of FMN against t-BOOH-induced oxidative injury in MC3T3-E1 cells, RNA-seq was performed in the t-BOOH and t-BOOH+FMN groups, with three independent biological replicates per group. Based on the cell viability results, 10 μM FMN was selected for transcriptomic analysis. Following differential expression analysis using an FDR threshold of 0.05 and an absolute log_2_ fold change cutoff of 1.5, a total of 45 DEGs were identified, comprising 13 upregulated and 32 downregulated genes (Figure 5A). Hierarchical clustering analysis revealed distinct expression patterns between the t-BOOH and t-BOOH+FMN groups (Figure 5B).

Notably, several AP-1-related genes, including *Fos*, *Junb*, *Fosb*, *Jund*, and *Jun*, were among the downregulated DEGs following FMN treatment (Figure 5A). GO enrichment analysis identified biological processes associated with osteoblast differentiation, ossification, OS responses, ROS responses, and intrinsic apoptotic signaling. The enriched cellular component terms included transcription regulator complexes and collagen-associated structures, whereas molecular function terms included transcriptional regulation and protein kinase regulatory activities (Figure 5C). KEGG enrichment analysis identified several pathways related to inflammatory and stress responses, including IL-17 signaling, tumor necrosis factor (TNF) signaling, and MAPK signaling, as well as the reactive oxygen species-related chemical carcinogenesis pathway (Figure 5D). Collectively, these transcriptomic findings suggest that FMN treatment is associated with alterations in AP-1-related gene expression and multiple biological processes involved in osteoblast function and cellular stress responses.

Based on the transcriptomic findings, Western blotting was performed to further assess the MAPK/AP-1 signaling axis at the protein level. t-BOOH markedly increased the phosphorylation levels of ERK1/2, JNK, and p38, whereas FMN treatment reduced the p-ERK1/2/ERK1/2, p-JNK/JNK and p-p38/p38 ratios, with greater effects observed at higher concentrations (Figure 5E–H). Consistent with the transcriptomic findings, t-BOOH markedly increased the expression of the AP-1 components c-Fos and c-Jun and enhanced c-Jun phosphorylation. FMN treatment significantly reduced the expression of c-Fos and c-Jun, as well as c-Jun phosphorylation, following t-BOOH exposure (Figure 5I–L). Together, these transcriptomic and protein-level findings indicate that FMN attenuates t-BOOH-induced activation of MAPK signaling and AP-1-related responses in MC3T3-E1 cells.

### 3.6. Reactivation of p38/JNK Signaling Attenuates the Antioxidant and Anti-Apoptotic Effects of FMN

To determine whether anisomycin could enhance p38/JNK signaling under the experimental conditions used, we first optimized its concentration and exposure time in MC3T3-E1 cells. Anisomycin reduced cell viability in a concentration- and time-dependent manner, whereas 0.16 μM anisomycin for 1 h caused relatively limited cytotoxicity while markedly increasing the phosphorylation of p38 and JNK (Supplementary Figure S3A–F). Higher concentrations did not produce a consistently greater increase in the p-p38/p38 or p-JNK/JNK ratios. Therefore, 0.16 μM anisomycin for 1 h was selected for subsequent rescue experiments. Under the FMN treatment condition, anisomycin significantly increased both the p-p38/p38 and p-JNK/JNK ratios compared with the t-BOOH+FMN group (Supplementary Figure S3G–I), indicating partial reactivation of p38/JNK signaling.

**Figure 6:**
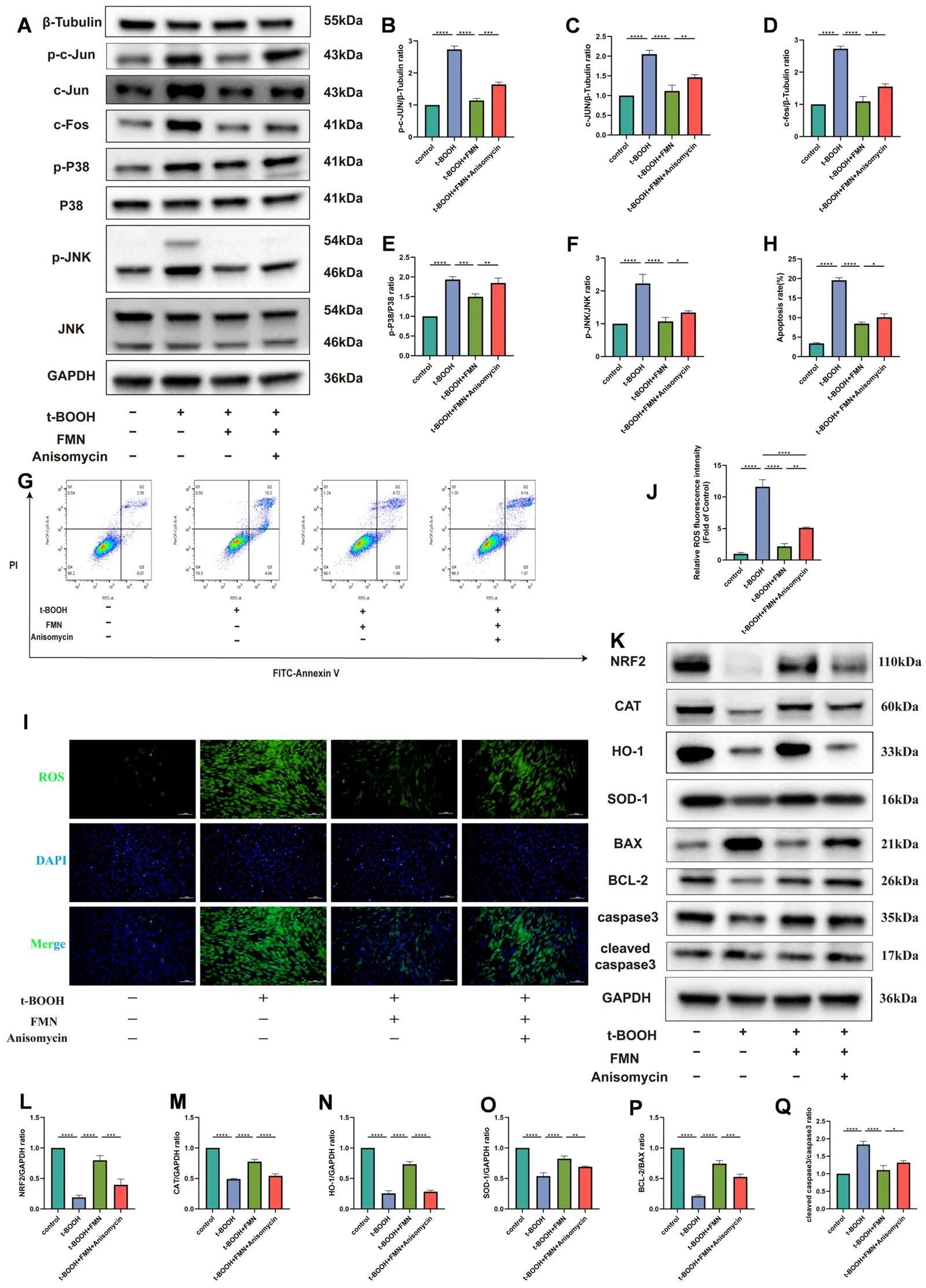
Reactivation of p38/JNK signaling attenuates the antioxidant and anti-apoptotic effects of FMN under oxidative stress. (**A**) Representative Western blots of p-c-Jun, c-Jun, c-Fos, p-p38, p38, p-JNK, and JNK under the indicated treatment conditions, with representative β-Tubulin and GAPDH bands shown. (**B**–**F**) Quantitative analysis of p-c-Jun/β-Tubulin (**B**), c-Jun/β-Tubulin (**C**), c-Fos/β-Tubulin (**D**), p-p38/p38 (**E**), and p-JNK/JNK (**F**). (**G**) Representative Annexin V-FITC/PI apoptosis plots. (**H**) Quantitative analysis of apoptosis rate. (**I**) Representative fluorescence images of intracellular ROS staining. Nuclei were counterstained with DAPI. (**J**) Quantitative analysis of ROS fluorescence intensity. (**K**) Representative Western blots of NRF2, CAT, HO-1, SOD1, BAX, BCL-2, caspase-3, and cleaved caspase-3, with GAPDH as the corresponding loading control. (**L**–**Q**) Quantitative analysis of NRF2/GAPDH (**L**), CAT/GAPDH (**M**), HO-1/GAPDH (**N**), SOD1/GAPDH (**O**), BCL-2/BAX ratio (**P**), and cleaved caspase-3/caspase-3 ratio (**Q**). Data are presented as mean ± SD from three independent biological experiments (*n* = 3). Statistical comparisons were performed using one-way ANOVA followed by Tukey’s multiple-comparisons test. ns, not significant; * *p* < 0.05, ** *p* < 0.01, *** *p* < 0.001, and **** *p* < 0.0001.

We next examined whether p38/JNK reactivation could attenuate the inhibitory effects of FMN on the MAPK/AP-1 axis. Under t-BOOH-induced OS, FMN markedly reduced the phosphorylation levels of p38 and JNK and decreased the expression of AP-1-related proteins, including c-Fos, c-Jun, and p-c-Jun. Anisomycin treatment significantly attenuated these effects, resulting in increased p38 and JNK phosphorylation and increased levels of AP-1-related proteins compared with the t-BOOH+FMN group (Figure 6A–F). Functionally, anisomycin also attenuated the effects of FMN on t-BOOH-induced OS and apoptosis. Flow cytometric analysis showed that FMN reduced the proportion of apoptotic cells, whereas anisomycin partially reversed this effect (Figure 6G,H). Similarly, ROS staining showed that anisomycin significantly increased intracellular ROS accumulation compared with the t-BOOH+FMN group (Figure 6I,J). Western blot analysis further showed that anisomycin attenuated the FMN-associated increases in NRF2, CAT, HO-1, and SOD1 expression (Figure 6K–O). Moreover, anisomycin decreased the BCL-2/BAX ratio and increased the cleaved caspase-3/caspase-3 ratio compared with the t-BOOH+FMN group (Figure 6K,P,Q). Collectively, these findings indicate that pharmacological reactivation of p38/JNK signaling attenuated the antioxidant and anti-apoptotic effects of FMN under t-BOOH-induced OS.

### 3.7. Anisomycin Attenuates FMN-Associated Preservation of Osteogenic Differentiation Under OS

**Figure 7:**
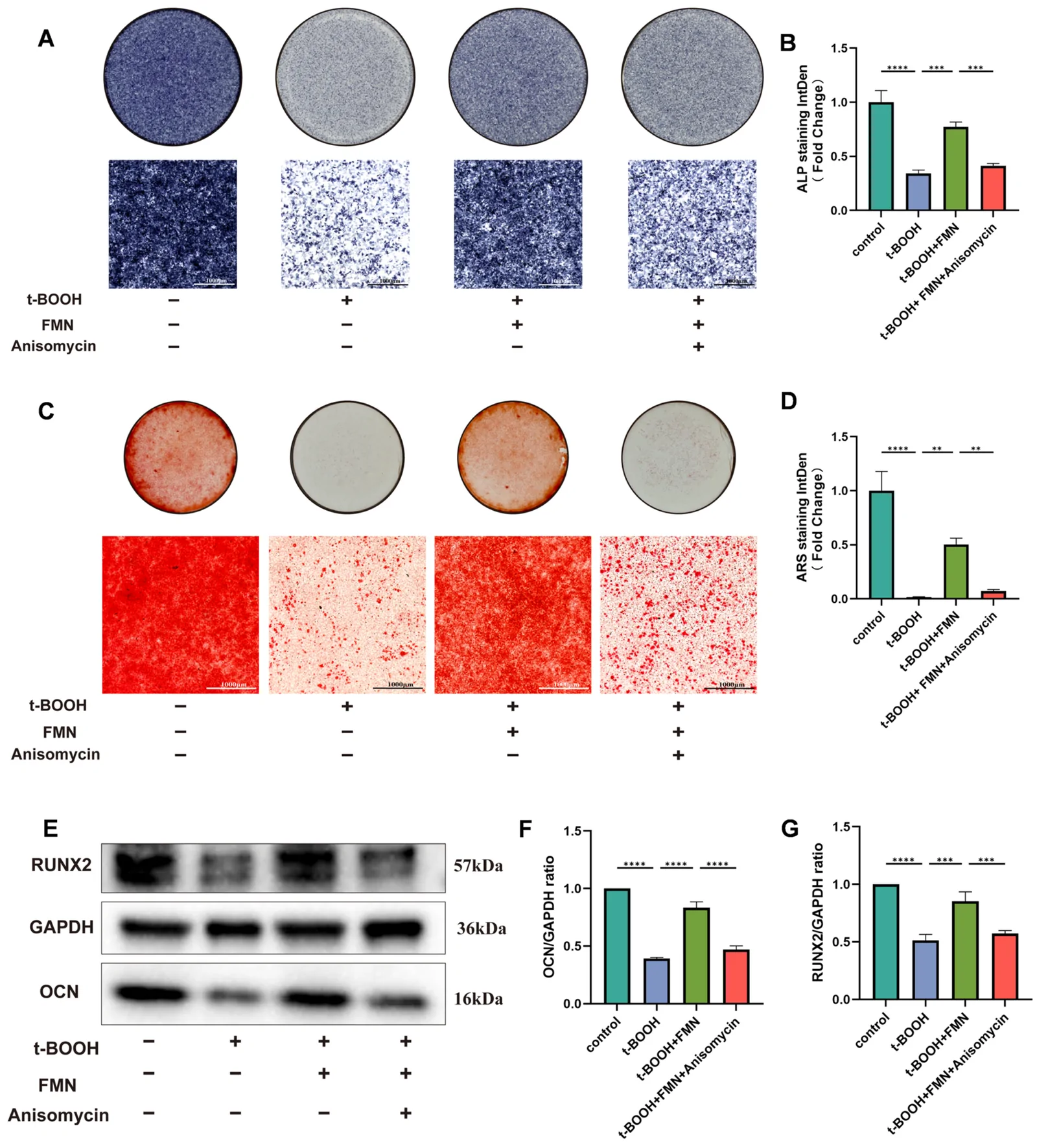
Anisomycin attenuates FMN-associated preservation of osteogenic differentiation under oxidative stress. (**A**) Representative ALP staining images. (**B**) Quantitative analysis of ALP staining intensity. (**C**) Representative ARS staining images. (**D**) Quantitative analysis of ARS staining intensity. (**E**) Western blot analysis of RUNX2 and OCN using GAPDH as the internal reference. (**F**,**G**) Quantitative analysis of OCN (**F**) and RUNX2 (**G**) protein levels. Data are presented as mean ± SD from three independent biological experiments (*n* = 3). Statistical comparisons were performed using one-way ANOVA followed by Tukey’s multiple-comparisons test. ns, not significant; ** *p* < 0.01, *** *p* < 0.001, and **** *p* < 0.0001.

We next investigated whether reactivation of p38/JNK signaling affected the ability of FMN to preserve osteogenic differentiation under OS. t-BOOH markedly reduced ALP staining intensity and mineralization capacity in MC3T3-E1 cells, whereas FMN treatment partially attenuated these effects. Anisomycin treatment significantly reduced the FMN-associated increases in ALP staining intensity and matrix mineralization, indicating that p38/JNK reactivation attenuated the preservation of osteogenic phenotypes by FMN under OS (Figure 7A–D). Consistent with the staining results, Western blot analysis showed that t-BOOH reduced the expression of the osteogenic markers RUNX2 and OCN, whereas FMN increased their expression. Anisomycin attenuated the FMN-induced increases in RUNX2 and OCN expression, further supporting an association between p38/JNK signaling and the pro-osteogenic effects of FMN (Figure 7E–G).

To further investigate whether NRF2-mediated antioxidant responses contribute to the protective effects of FMN, ML385 was used to inhibit NRF2 activity. Compared with the t-BOOH+FMN group, ML385 reduced the expression of NRF2 and antioxidant-related proteins, including CAT and SOD1, and decreased the expression of the osteogenic markers RUNX2 and OCN (Supplementary Figure S4A,C–G). In addition, ML385 reduced the BCL-2/BAX ratio and increased the cleaved caspase-3/caspase-3 ratio, suggesting partial attenuation of the anti-apoptotic effects of FMN under OS (Supplementary Figure S4B,H,I). Collectively, these pharmacological findings are consistent with the involvement of NRF2-associated antioxidant responses and p38/JNK signaling in the protective effects observed following FMN treatment under OS.

### 3.8. FMN Suppresses MAPK/AP-1 Activation and Reduces Apoptosis in Bone Tissue of OVX Mice

**Figure 8:**
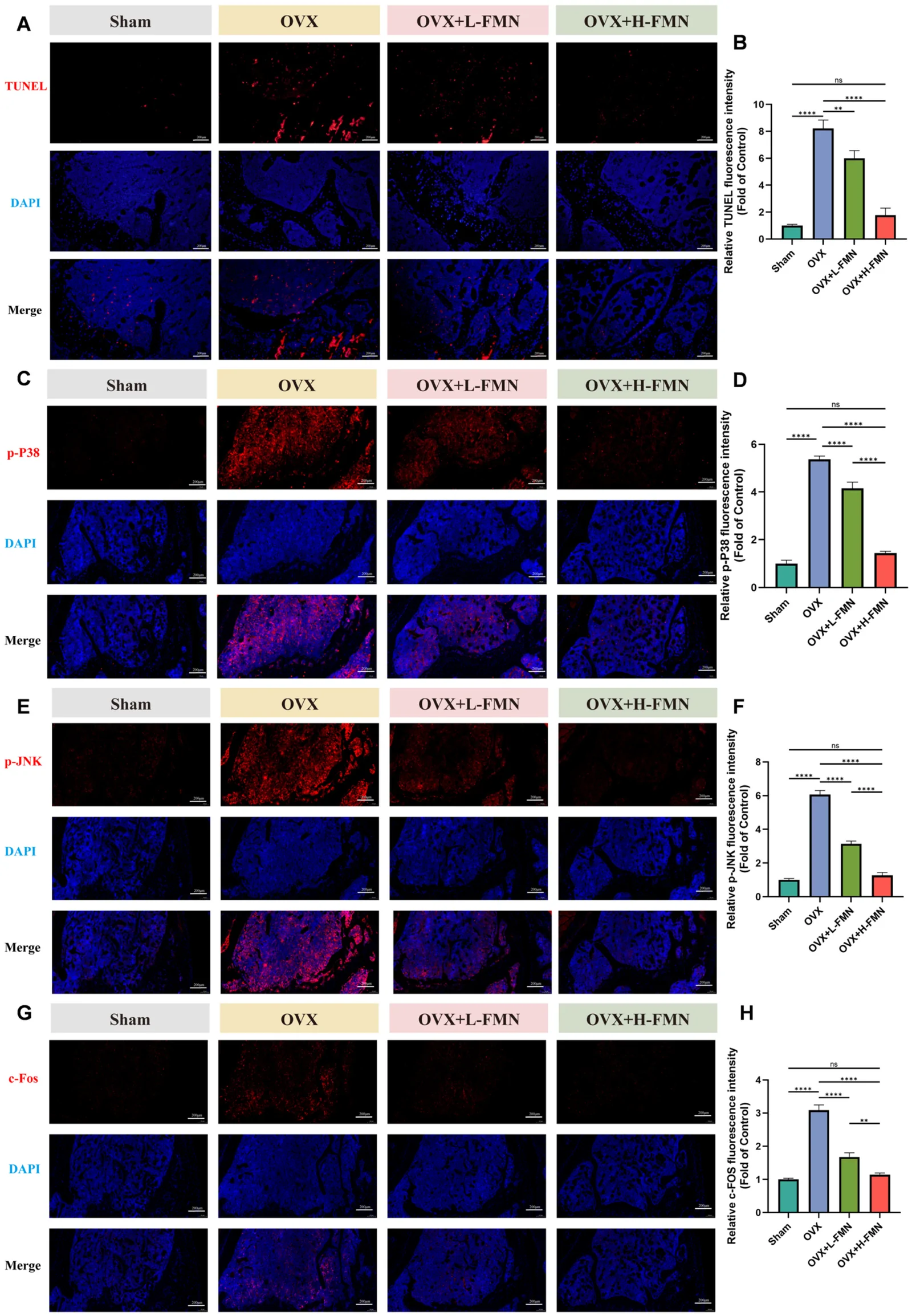
FMN reduces apoptosis-associated TUNEL signals and MAPK/AP-1-related immunofluorescence in OVX mice. (**A**) Representative TUNEL staining images of distal femoral sections. (**B**) Quantitative analysis of TUNEL fluorescence intensity. (**C**,**E**,**G**) Representative immunofluorescence staining images of p-p38 (**C**), p-JNK (**E**), and c-Fos (**G**) in distal femoral sections. (**D**,**F**,**H**) Quantitative analysis of p-p38 (**D**), p-JNK (**F**), and c-Fos (**H**) fluorescence intensity. Scale bars = 200 μm. Data are presented as mean ± SD from three independent mice per group (*n* = 3). Statistical comparisons were performed using one-way ANOVA followed by Tukey’s multiple-comparisons test. ns, not significant; ** *p* < 0.01 and **** *p* < 0.0001.

To further assess the in vivo relevance of the cellular findings, immunofluorescence staining was performed on distal femoral sections from OVX mice. TUNEL staining revealed markedly increased fluorescence intensity in the trabecular bone region of OVX mice compared with the Sham group. In contrast, FMN treatment decreased TUNEL-positive signals, with the most pronounced reduction observed in the OVX+H-FMN group (Figure 8A,B). These findings indicate that FMN attenuates OVX-induced apoptosis in bone tissue.

We next examined whether FMN modulated MAPK/AP-1 signaling in vivo. Immunofluorescence staining showed that the fluorescence intensities of p-p38 and p-JNK were markedly increased in the distal femur of OVX mice compared with the Sham group, consistent with increased p38/JNK signaling activity following estrogen deficiency. FMN treatment reduced the fluorescence intensities of p-p38 and p-JNK, particularly at the higher dose (Figure 8C–F). Consistent with these findings, the AP-1 component c-Fos showed increased expression in the OVX group, whereas FMN treatment reduced c-Fos expression in bone tissue (Figure 8G,H). Together, these in vivo findings are consistent with the cellular results and support an association between FMN treatment, reduced p38/JNK signaling and decreased apoptosis in the bone tissue of OVX mice.

## 4. Discussion

OP is a systemic skeletal disorder generally categorized into primary and secondary forms, with primary OP mainly including postmenopausal and age-related types [35]. PMOP is mainly associated with estrogen deficiency, which promotes increased bone turnover and gradual loss of bone mass. Although the pathogenesis of OP is multifactorial, OS, inflammation, mitochondrial dysfunction, aging and expansion of bone marrow adipose tissue are recognized as important contributors to disrupted bone homeostasis [36,37,38]. In particular, estrogen deficiency can disrupt redox homeostasis by impairing antioxidant defenses and increasing the susceptibility of bone cells to oxidative injury [39,40]. Estrogen-related receptor signaling has also been implicated in cellular redox sensing and OS responses [41]. Consistent with these observations, postmenopausal women exhibit lower circulating levels of antioxidant biomarkers, such as SOD, glutathione peroxidase, and total antioxidant capacity, accompanied by elevated levels of oxidative products [42]. Serum lipid peroxide levels have also been reported to correlate inversely with lumbar spine bone mineral density, further supporting an association between oxidative imbalance and postmenopausal bone loss [43]. Similar alterations in OS markers have been observed in OVX animal models and are associated with reduced bone mineral density [44,45]. Excessive OS can promote osteoblast apoptosis and impair osteoblast function, thereby contributing to an imbalance between bone formation and resorption [46]. Therefore, strategies that mitigate OS and preserve osteoblast function may represent a promising therapeutic approach for PMOP.

FMN is a phytoestrogen with antioxidant, anti-inflammatory and estrogenic properties that may contribute to its potential osteoprotective effects [47,48,49]. Previous research has demonstrated that FMN suppresses osteoclast differentiation and reduces the expression of osteoclast-specific genes through regulation of the nuclear factor kappa B, MAPK, and AP-1 signaling pathways, thereby attenuating osteolytic bone loss under various pathological conditions [50,51]. However, previous studies of the skeletal effects of FMN have predominantly focused on osteoclasts, whereas its effects on osteoblasts under OS remain less well characterized. Given that estrogen deficiency promotes oxidative damage and impairs osteoblast function, the antioxidant activity of FMN may provide an additional mechanism underlying its anti-osteoporotic effects [52]. In the present study, FMN attenuated trabecular bone loss in the distal femoral metaphysis of OVX mice. Micro-CT and histological analyses showed that FMN partially preserved trabecular microarchitecture, with greater improvements generally observed in the high-dose FMN group. For several microarchitectural parameters, high-dose FMN produced changes that were descriptively similar to those observed with E_2_ treatment. However, no formal equivalence or non-inferiority analysis was performed, and these observations should not be interpreted as evidence of equivalent efficacy. In parallel, FMN reduced serum MDA levels and increased SOD activity, consistent with an improvement in systemic redox status following OVX. Previous pharmacokinetic studies have indicated that FMN is absorbed through the intestine and undergoes metabolism primarily in the liver and kidneys [53]. In the present study, no apparent histopathological abnormalities were observed in major organs after FMN administration. Collectively, these findings suggest that FMN attenuates OVX-induced trabecular bone loss and systemic oxidative imbalance and was not associated with apparent histopathological abnormalities in major organs under the experimental conditions used.

Excessive ROS accumulation can disrupt mitochondrial homeostasis, promote apoptosis and impair osteoblast function. To model oxidative injury in vitro, MC3T3-E1 cells were exposed to t-BOOH, a relatively stable organic peroxide that can induce sustained OS [54]. t-BOOH treatment reduced cell viability, increased intracellular ROS accumulation and apoptosis, induced mitochondrial ultrastructural damage, and impaired osteogenic differentiation and matrix mineralization. FMN attenuated these alterations, with greater effects generally observed at higher concentrations, suggesting that FMN protects osteoblasts against oxidative injury while preserving their osteogenic capacity. Consistent with its antioxidant effects, FMN increased the expression of NRF2 and NRF2-associated antioxidant proteins, including HO-1, CAT, GPX1, and SOD1. NRF2 serves as a major transcriptional regulator of cellular antioxidant defense, promoting the expression of genes that maintain redox homeostasis and protect against oxidative damage [55]. Pharmacological inhibition of NRF2 with ML385 reduced the expression of NRF2, CAT and SOD1, together with the osteogenic markers RUNX2 and OCN, in FMN-treated cells. Moreover, ML385 decreased the BCL-2/BAX ratio and increased the cleaved caspase-3/caspase-3 ratio, indicating attenuation of the anti-apoptotic effects of FMN. Collectively, these findings suggest that NRF2-associated antioxidant responses contribute to the antioxidant, anti-apoptotic and pro-osteogenic effects of FMN under t-BOOH-induced OS.

Network pharmacology and enrichment analyses highlighted MAPK signaling and OS-related processes as candidate pathways associated with the effects of FMN. Molecular docking predicted favorable interactions between FMN and MAPK1/ERK2, MAPK8/JNK1 and MAPK14/p38α. Subsequent 100 ns molecular dynamics simulations showed relatively stable RMSD profiles, limited fluctuations in Rg and distinct low-energy conformational basins, providing additional computational support for the stability of the predicted FMN–MAPK complexes. These computational findings support the structural plausibility of the predicted interactions but do not establish direct target engagement or biochemical inhibition. Consistent with these predictions, transcriptomic analysis showed that FMN markedly downregulated AP-1-related genes, including *Fos, Junb, Fosb, Jund and Jun*. AP-1 is composed primarily of Jun and Fos family proteins and is regulated by stress-responsive MAPK signaling, with JNK-mediated phosphorylation of c-Jun representing an important mechanism of AP-1 activation [56,57]. Correspondingly, FMN reduced t-BOOH-induced phosphorylation of ERK1/2, p38 and JNK, while decreasing the expression of c-Fos and c-Jun and the phosphorylation of c-Jun. Collectively, these findings support the involvement of the MAPK/AP-1 signaling axis in the effects of FMN under OS and suggest that FMN attenuates t-BOOH-induced activation of this axis.

To further examine whether modulation of MAPK/AP-1 signaling was associated with the protective effects of FMN, we performed pharmacological pathway-modulation experiments using anisomycin, which increased p38 and JNK phosphorylation under the experimental conditions used [58]. Anisomycin attenuated the FMN-associated reductions in c-Fos and c-Jun expression and c-Jun phosphorylation and increased intracellular ROS accumulation and apoptosis compared with FMN treatment alone. In addition, anisomycin partially diminished the FMN-associated preservation of ALP staining intensity, matrix mineralization, and the expression of the osteogenic markers RUNX2 and OCN. Taken together, these findings are consistent with an involvement of stress-responsive p38/JNK–AP-1 signaling in the cellular effects associated with FMN treatment. However, these pharmacological experiments do not establish pathway-specific causality or demonstrate that MAPK/AP-1 signaling is the exclusive mediator of FMN action.

At the tissue level, immunofluorescence staining of the distal femur revealed that OVX enhanced the signals of p-p38, p-JNK, and the AP-1 component c-Fos. In contrast, FMN treatment suppressed these fluorescence signals, with more pronounced effects generally observed in the high-dose FMN group. These findings were consistent with the suppression of p38/JNK–AP-1 signaling observed in the t-BOOH-induced cellular model. TUNEL staining further showed that FMN reduced apoptosis-associated fluorescence intensity in the trabecular bone region of OVX mice. Collectively, these in vivo findings support an association between reduced p38/JNK–AP-1-related signaling and attenuation of apoptosis-associated changes following FMN treatment, extending the cellular observations to the OVX model.

Nevertheless, several limitations of this study should be acknowledged. First, although our findings support the involvement of MAPK/AP-1 signaling in the protective effects of FMN, its precise molecular targets remain incompletely defined. Molecular docking and molecular dynamics simulations suggested potential interactions between FMN and MAPK1/ERK2, MAPK8/JNK1, and MAPK14/p38α; however, these computational predictions do not establish direct target engagement. Further biochemical binding or kinase activity assays are needed to determine whether FMN directly interacts with specific MAPK family members or modulates these pathways through upstream signaling components. Second, the pharmacological experiments relied on anisomycin to reactivate p38/JNK signaling and ML385 to inhibit NRF2. Because anisomycin also inhibits protein synthesis and can independently induce cellular stress, some of the observed changes in protein expression, ROS accumulation, apoptosis, and osteogenic outcomes may not be attributable solely to p38/JNK activation. Moreover, pharmacological modulation alone cannot establish pathway-specific causality or determine the relative contributions of MAPK/AP-1 and NRF2 signaling to the protective effects of FMN. Third, this study primarily focused on osteoblast-associated mechanisms, whereas bone homeostasis depends on coordinated interactions between osteoblasts and osteoclasts. Although previous studies have suggested that FMN modulates osteoclast differentiation and function, its contribution to the bone-protective effects observed in the present study remains uncertain. Similarly, the potential contribution of FMN’s estrogenic properties to its effects in OVX mice cannot be distinguished from its antioxidant and osteoblast-associated activities based on the current experimental findings. Fourth, although FMN modulated ERK1/2, p38, and JNK phosphorylation, the functional contribution of ERK1/2 was not investigated in depth. Because ERK1/2 can also regulate AP-1 activity, potential crosstalk and compensatory interactions between ERK1/2 and the p38/JNK branches of MAPK signaling warrant further investigation. Fifth, because FMN improved cell viability under OS, the observed increases in ALP staining intensity and matrix mineralization may partly reflect improved cell survival or cell number rather than enhanced osteogenic differentiation alone. Further studies are required to distinguish these effects. Finally, the relatively small sample size and the use of MC3T3-E1 cells and an OVX mouse model may limit the generalizability of the findings. In addition, no a priori sample-size calculation was performed and no single primary outcome was prespecified; therefore, the present study should be considered exploratory. Effect sizes and corresponding confidence intervals were not systematically reported, which limits assessment of the magnitude and precision of the observed effects. Although previous studies have investigated the pharmacokinetic properties of FMN, its pharmacokinetic profile was not evaluated under the experimental conditions used here. In addition, bone biomechanical properties and long-term safety were not assessed. Further investigations addressing these limitations are needed to establish the broader relevance of the observed effects and clarify the therapeutic potential of FMN.

## 5. Conclusions

**Figure 9:**
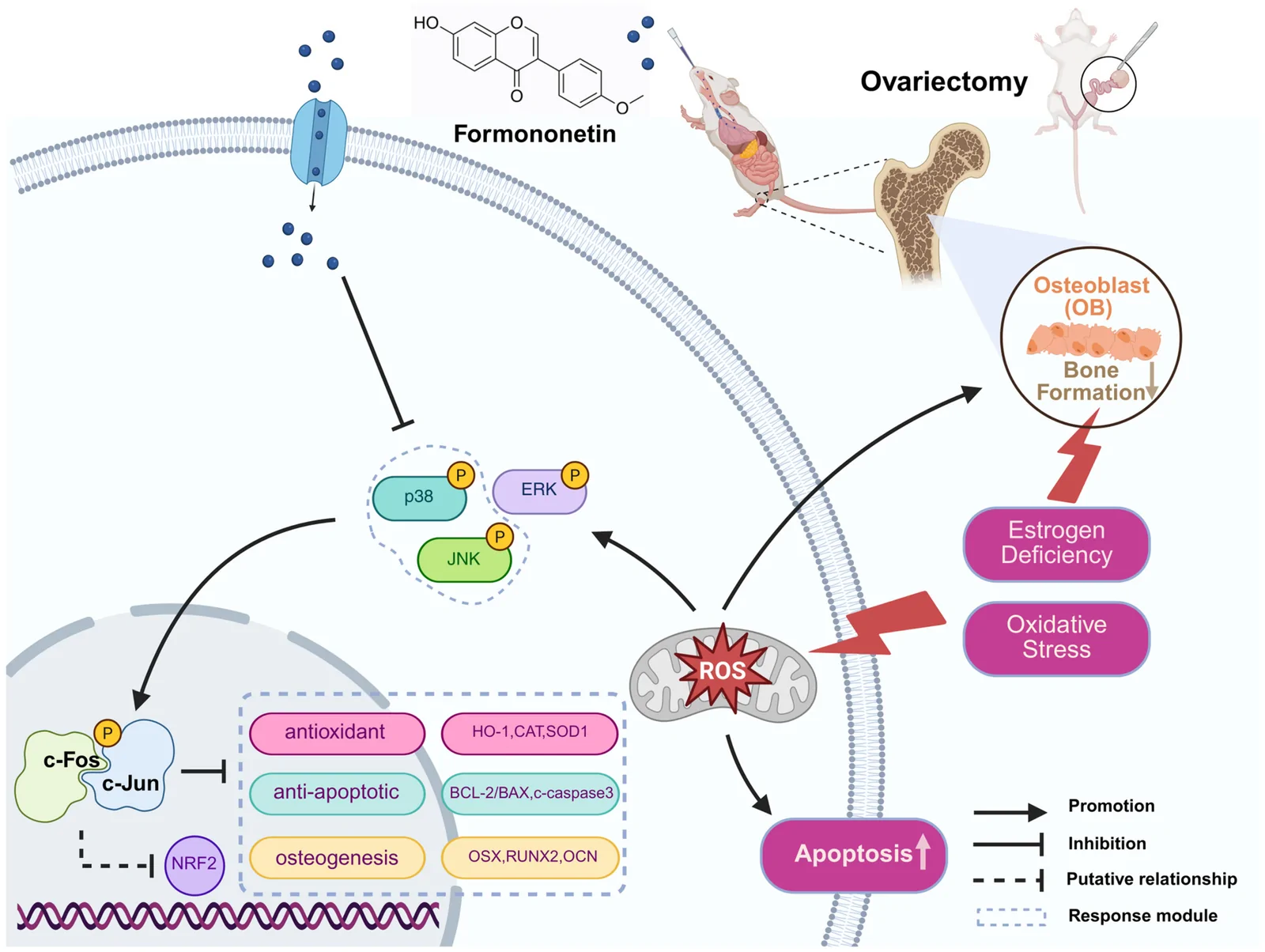
Schematic representation of the signaling changes associated with FMN treatment in the present study. FMN treatment was associated with reduced MAPK/AP-1 signaling and enhanced NRF2-associated antioxidant responses, accompanied by reduced osteoblast apoptosis and preservation of osteogenic function under OS. The dashed inhibitory connection between AP-1 and NRF2 represents a putative relationship that was not directly examined in the present study. Created in BioRender. Shuhua, L. (2026) https://BioRender.com/okewrsd.

In this study, FMN attenuated OVX-associated trabecular bone deterioration in mice and protected MC3T3-E1 cells against t-BOOH-induced OS, apoptosis, and impaired osteogenic differentiation. FMN treatment was accompanied by reduced activation of MAPK/AP-1-related signaling and enhanced NRF2-associated antioxidant responses. Pharmacological modulation with anisomycin and ML385 further supported the involvement of these signaling responses in the cellular effects associated with FMN treatment, although these experiments do not establish pathway-specific causality or direct molecular targeting. Collectively, these findings identify MAPK/AP-1 and NRF2-associated signaling as candidate pathways linked to the protective effects of FMN and provide a basis for further mechanistic investigation. A schematic summary of the signaling changes associated with FMN treatment is shown in Figure 9.

## Data Availability

The original contributions presented in this study are included in the article/Supplementary Materials. The raw RNA-seq data generated in this study have been deposited in the NCBI Sequence Read Archive (SRA) under BioProject accession number PRJNA1533408. The raw data supporting the conclusions of this article will be made available by the authors on request.

## Supplementary Materials

The following supporting information can be downloaded at https://www.mdpi.com/article/10.3390/antiox15101348/s1. Supplementary Figure S1. FMN preserves RUNX2 expression in the distal femur and shows no obvious histological toxicity in major organs. (**A**) Representative RUNX2 immunofluorescence staining images of distal femoral sections. RUNX2 is shown in red, and nuclei were counterstained with DAPI in blue. (**B**) Quantitative analysis of RUNX2 fluorescence intensity. (**C**) Representative H&E staining images of the heart, liver, spleen, lung, and kidney. Scale bars = 200 μm. (**D**) Representative gross photographs of uterine tissues from Sham and OVX mice, showing a visible reduction in uterine size following ovariectomy. A metric ruler is included as a size reference. Data are presented as mean ± SD from three independent mice per group (*n* = 3). Statistical comparisons were performed using one-way ANOVA followed by Tukey’s multiple-comparisons test. ns, not significant; * *p* < 0.05, *** *p* < 0.001, and **** *p* < 0.0001. Supplementary Figure S2. Molecular docking and molecular dynamics simulation of the FMN–MAPK1 complex. (**A**) Molecular docking analysis of FMN with MAPK1, including the overall docking conformation, enlarged binding pocket, and two-dimensional interaction diagram. (**B**) Residue energy decomposition analysis of the FMN–MAPK1 complex. (**C**) Root mean square deviation (RMSD) plot of the FMN–MAPK1 complex during 100 ns molecular dynamics simulation. (**D**,**E**) Gibbs free energy landscape of the FMN–MAPK1 complex. (**F**) Radius of gyration (Rg) analysis of the FMN–MAPK1, FMN–MAPK8, and FMN–MAPK14 complexes. Supplementary Figure S3. Optimization and validation of anisomycin-mediated p38/JNK pathway activation. (**A**–**C**) Cell viability of MC3T3-E1 cells after treatment with anisomycin at 0.032, 0.16, 0.8, 4, and 20 μM for 1 h (**A**), 2 h (**B**), and 3 h (**C**), respectively. (**D**) Representative Western blot analysis of p-JNK, JNK, p-p38, and p38 after treatment with anisomycin at 0.16, 0.8, and 4 μM for 1 h, with GAPDH shown as the loading control. (**E**,**F**) Quantitative analysis of the p-p38/p38 ratio (**E**) and p-JNK/JNK ratio (**F**). (**G**) Representative Western blot analysis of p-JNK, JNK, p-p38, and p38 under the indicated t-BOOH, FMN, and anisomycin treatment conditions. (**H**,**I**) Quantitative analysis of the p-JNK/JNK ratio (**H**) and p-p38/p38 ratio (**I**). Data are presented as mean ± SD from three independent biological experiments (*n* = 3). Statistical comparisons were performed using one-way ANOVA followed by Tukey’s multiple-comparisons test. ns, not significant; * *p* < 0.05, ** *p* < 0.01, *** *p* < 0.001, and **** *p* < 0.0001. Supplementary Figure S4. NRF2 inhibition attenuates the antioxidant, anti-apoptotic, and pro-osteogenic effects of FMN under oxidative stress. (**A**) Western blot analysis of NRF2, CAT, RUNX2, SOD1, and OCN in t-BOOH+FMN-treated MC3T3-E1 cells with or without ML385, using GAPDH as the internal reference. (**B**) Western blot analysis of caspase-3, cleaved caspase-3, BCL-2, and BAX using GAPDH as the internal reference. (**C**–**G**) Quantitative analysis of NRF2 (**C**), CAT (**D**), SOD1 (**E**), RUNX2 (**F**), and OCN (**G**) protein levels. (**H**,**I**) Quantitative analysis of BCL-2/BAX ratio (**H**) and cleaved caspase-3/caspase-3 ratio (**I**). Data are presented as mean ± SD from three independent biological experiments (*n* = 3). Statistical comparisons were performed using an unpaired two-tailed Student’s *t*-test. ns, not significant; * *p* < 0.05, ** *p* < 0.01, and *** *p* < 0.001. ML385, NRF2 inhibitor. Supplementary Table S1. The target of FMN (*n* = 166). Supplementary Table S2. Drug–disease intersection targets (*n* = 139). Supplementary Table S3. Molecular docking parameters for the FMN–MAPK complexes.

## Abbreviations

The following abbreviations are used in this manuscript:

ALP	Alkaline phosphatase
AP-1	Activator protein 1
ARS	Alizarin Red S
BAX	BCL-2-associated X protein
BCL-2	B-cell lymphoma 2
BV/TV	Bone volume fraction
CAT	Catalase
DAPI	4′,6-diamidino-2-phenylindole
DEGs	Differentially expressed genes
E_2_	Estradiol valerate
ERK	Extracellular signal-regulated kinase
FDR	False discovery rate
FMN	Formononetin
GAPDH	Glyceraldehyde-3-phosphate dehydrogenase
GO	Gene Ontology
GPX1	Glutathione peroxidase 1
H&E	Hematoxylin and eosin
HO-1	Heme oxygenase 1
JNK	c-Jun N-terminal kinase
KEGG	Kyoto Encyclopedia of Genes and Genomes
MAPK	Mitogen-activated protein kinase
MDA	Malondialdehyde
micro-CT	Micro-computed tomography
NRF2	Nuclear factor erythroid 2-related factor 2
OCN	Osteocalcin
OP	Osteoporosis
OS	Oxidative stress
OSX	Osterix
OVX	Ovariectomy
PMOP	Postmenopausal osteoporosis
PPI	Protein–protein interaction
ROS	Reactive oxygen species
RUNX2	Runt-related transcription factor 2
SMI	Structure model index
SOD	Superoxide dismutase
SOD1	Superoxide dismutase 1
t-BOOH	tert-butyl hydroperoxide
Tb.N	Trabecular number
Tb.Sp	Trabecular separation
Tb.Th	Trabecular thickness
TUNEL	Terminal deoxynucleotidyl transferase dUTP nick-end labeling

## References

[1] Compston J.E., McClung M.R., Leslie W.D. (2019). Osteoporosis. Lancet.

[2] Walker M.D., Shane E. (2023). Postmenopausal Osteoporosis. N. Engl. J. Med..

[3] Morin S.N., Leslie W.D., Schousboe J.T. (2025). Osteoporosis: A Review. JAMA.

[4] Kim H., Lee K., Kim J.M., Kim M.Y., Kim J.R., Lee H.W., Chung Y.W., Shin H.I., Kim T., Park E.S. (2021). Selenoprotein W ensures physiological bone remodeling by preventing hyperactivity of osteoclasts. Nat. Commun..

[5] Riegger J., Schoppa A., Ruths L., Haffner-Luntzer M., Ignatius A. (2023). Oxidative stress as a key modulator of cell fate decision in osteoarthritis and osteoporosis: A narrative review. Cell Mol. Biol. Lett..

[6] Yu B., Wang C.Y. (2022). Osteoporosis and periodontal diseases—An update on their association and mechanistic links. Periodontology 2000.

[7] Marcucci G., Domazetovic V., Nediani C., Ruzzolini J., Favre C., Brandi M.L. (2023). Oxidative Stress and Natural Antioxidants in Osteoporosis: Novel Preventive and Therapeutic Approaches. Antioxidants.

[8] Li W., Jiang W.S., Su Y.R., Tu K.W., Zou L., Liao C.R., Wu Q., Wang Z.H., Zhong Z.M., Chen J.T. (2023). PINK1/Parkin-mediated mitophagy inhibits osteoblast apoptosis induced by advanced oxidation protein products. Cell Death Dis..

[9] Jin W., Zhu X., Yao F., Xu X., Chen X., Luo Z., Zhao D., Li X., Leng X., Sun L. (2020). Cytoprotective effect of Fufang Lurong Jiangu capsule against hydrogen peroxide-induced oxidative stress in bone marrow stromal cell-derived osteoblasts through the Nrf2/HO-1 signaling pathway. Biomed. Pharmacother..

[10] Ali D., Chen L., Kowal J.M., Okla M., Manikandan M., AlShehri M., AlMana Y., AlObaidan R., AlOtaibi N., Hamam R. (2020). Resveratrol inhibits adipocyte differentiation and cellular senescence of human bone marrow stromal stem cells. Bone.

[11] Chang L., Karin M. (2001). Mammalian MAP kinase signalling cascades. Nature.

[12] Chen Z., Gibson T.B., Robinson F., Silvestro L., Pearson G., Xu B., Wright A., Vanderbilt C., Cobb M.H. (2001). MAP kinases. Chem. Rev..

[13] Gehart H., Kumpf S., Ittner A., Ricci R. (2010). MAPK signalling in cellular metabolism: Stress or wellness?. EMBO Rep..

[14] Trop-Steinberg S., Azar Y. (2017). AP-1 Expression and its Clinical Relevance in Immune Disorders and Cancer. Am. J. Med. Sci..

[15] Fischer B.M., Voynow J.A., Ghio A.J. (2015). COPD: Balancing oxidants and antioxidants. Int. J. Chron. Obstruct. Pulmon. Dis..

[16] Jope R.S., Zhang L., Song L. (2000). Peroxynitrite modulates the activation of p38 and extracellular regulated kinases in PC12 cells. Arch. Biochem. Biophys..

[17] Mercer B.A., Kolesnikova N., Sonett J., D’Armiento J. (2004). Extracellular regulated kinase/mitogen activated protein kinase is up-regulated in pulmonary emphysema and mediates matrix metalloproteinase-1 induction by cigarette smoke. J. Biol. Chem..

[18] Gupta S., Campbell D., Derijard B., Davis R.J. (1995). Transcription factor ATF2 regulation by the JNK signal transduction pathway. Science.

[19] Zhou Z.W., Zhu X.Y., Li S.Y., Lin S.E., Zhu Y.H., Ji K., Chen J.J. (2023). Formononetin Inhibits Mast Cell Degranulation to Ameliorate Compound 48/80-Induced Pseudoallergic Reactions. Molecules.

[20] Almatroodi S.A., Almatroudi A., Khan A.A., Rahmani A.H. (2023). Potential Therapeutic Targets of Formononetin, a Type of Methoxylated Isoflavone, and Its Role in Cancer Therapy through the Modulation of Signal Transduction Pathways. Int. J. Mol. Sci..

[21] Ding M., Bao Y., Liang H., Zhang X., Li B., Yang R., Zeng N. (2024). Potential mechanisms of formononetin against inflammation and oxidative stress: A review. Front. Pharmacol..

[22] Liu G., Zhao W., Bai J., Cui J., Liang H., Lu B. (2021). Formononetin protects against concanavalin-A-induced autoimmune hepatitis in mice through its anti-apoptotic and anti-inflammatory properties. Biochem. Cell Biol..

[23] Luo L., Zhou J., Zhao H., Fan M., Gao W. (2019). The anti-inflammatory effects of formononetin and ononin on lipopolysaccharide-induced zebrafish models based on lipidomics and targeted transcriptomics. Metabolomics.

[24] Mendonca M., Ribeiro A., Lima A.K., Bezerra G.B., Pinheiro M.S., Albuquerque-Junior R., Gomes M.Z., Padilha F.F., Thomazzi S.M., Novellino E. (2020). Red Propolis and Its Dyslipidemic Regulator Formononetin: Evaluation of Antioxidant Activity and Gastroprotective Effects in Rat Model of Gastric Ulcer. Nutrients.

[25] Sugimoto M., Ko R., Goshima H., Koike A., Shibano M., Fujimori K. (2021). Formononetin attenuates H(2)O(2)-induced cell death through decreasing ROS level by PI3K/Akt-Nrf2-activated antioxidant gene expression and suppressing MAPK-regulated apoptosis in neuronal SH-SY5Y cells. Neurotoxicology.

[26] Xiao Q., Huang J., Zhu X., Shi M., Chen L., Chen L., Liu X., Liu R., Zhong Y. (2024). Formononetin ameliorates dextran sulfate sodium-induced colitis via enhancing antioxidant capacity, promoting tight junction protein expression and reshaping M1/M2 macrophage polarization balance. Int. Immunopharmacol..

[27] Xu H.T., Zheng Q., Tai Z.G., Jiang W.C., Xie S.Q., Luo Y., Fei X.Y., Luo Y., Ma X., Kuai L. (2024). Formononetin attenuates psoriasiform inflammation by regulating interferon signaling pathway. Phytomedicine.

[28] Liu M., Yang C., Peng X., Zheng S., He H., Wang W., Li Y. (2025). Formononetin suppresses colitis-associated colon cancer by targeting lipid synthesis and mTORC2/Akt signaling. Phytomedicine.

[29] Gautam J., Khedgikar V., Kushwaha P., Choudhary D., Nagar G.K., Dev K., Dixit P., Singh D., Maurya R., Trivedi R. (2017). Formononetin, an isoflavone, activates AMP-activated protein kinase/beta-catenin signalling to inhibit adipogenesis and rescues C57BL/6 mice from high-fat diet-induced obesity and bone loss. Br. J. Nutr..

[30] Kaczmarczyk-Sedlak I., Wojnar W., Zych M., Ozimina-Kaminska E., Taranowicz J., Siwek A. (2013). Effect of formononetin on mechanical properties and chemical composition of bones in rats with ovariectomy-induced osteoporosis. Evid. Based Complement. Altern. Med..

[31] Yan X.J., Wang Z.J., Wang H., Wei M.Z., Chen Y.C., Zhao Y.L., Luo X.D. (2024). Formononetin Derivative for Osteoporosis by Simultaneous Regulating Osteoblast and Osteoclast. J. Nat. Prod..

[32] Singh K.B., Dixit M., Dev K., Maurya R., Singh D. (2017). Formononetin, a methoxy isoflavone, enhances bone regeneration in a mouse model of cortical bone defect. Br. J. Nutr..

[33] Abraham M.J., Murtola T., Schulz R., Páll S., Smith J.C., Hess B., Lindahl E. (2015). GROMACS: High performance molecular simulations through multi-level parallelism from laptops to supercomputers. SoftwareX.

[34] Larsen K.L., Piana S., Palmo K., Maragakis P., Klepeis J.L., Dror R.O., Shaw D.E. (2010). Improved side-chain torsion potentials for the Amber ff99SB protein force field. Proteins.

[35] Brown C. (2017). Osteoporosis: Staying strong. Nature.

[36] Fischer V., Haffner-Luntzer M. (2022). Interaction between bone and immune cells: Implications for postmenopausal osteoporosis. Semin. Cell Dev. Biol..

[37] Li J., Chen X., Lu L., Yu X. (2020). The relationship between bone marrow adipose tissue and bone metabolism in postmenopausal osteoporosis. Cytokine Growth Factor Rev..

[38] Luo J., Li L., Shi W., Xu K., Shen Y., Dai B. (2025). Oxidative stress and inflammation: Roles in osteoporosis. Front. Immunol..

[39] Almeida M., Han L., Martin-Millan M., Plotkin L.I., Stewart S.A., Roberson P.K., Kousteni S., O’Brien C.A., Bellido T., Parfitt A.M. (2007). Skeletal involution by age-associated oxidative stress and its acceleration by loss of sex steroids. J. Biol. Chem..

[40] Solar F.V., Cipolletti M., Ascenzi P., Marino M., Fiocchetti M. (2020). Neuroglobin As Key Mediator in the 17beta-Estradiol-Induced Antioxidant Cell Response to Oxidative Stress. Antioxid. Redox Signal..

[41] Vernier M., Dufour C.R., McGuirk S., Scholtes C., Li X., Bourmeau G., Kuasne H., Park M., St-Pierre J., Audet-Walsh E. (2020). Estrogen-related receptors are targetable ROS sensors. Genes Dev..

[42] Zhou Q., Zhu L., Zhang D., Li N., Li Q., Dai P., Mao Y., Li X., Ma J., Huang S. (2016). Oxidative Stress-Related Biomarkers in Postmenopausal Osteoporosis: A Systematic Review and Meta-Analyses. Dis. Markers.

[43] Cervellati C., Bonaccorsi G., Cremonini E., Bergamini C.M., Patella A., Castaldini C., Ferrazzini S., Capatti A., Picarelli V., Pansini F.S. (2013). Bone mass density selectively correlates with serum markers of oxidative damage in post-menopausal women. Clin. Chem. Lab. Med..

[44] Shi C., Wu J., Yan Q., Wang R., Miao D. (2015). Bone marrow ablation demonstrates that estrogen plays an important role in osteogenesis and bone turnover via an antioxidative mechanism. Bone.

[45] Geng Q., Gao H., Yang R., Guo K., Miao D. (2019). Pyrroloquinoline Quinone Prevents Estrogen Deficiency-Induced Osteoporosis by Inhibiting Oxidative Stress and Osteocyte Senescence. Int. J. Biol. Sci..

[46] Chandra A., Rajawat J. (2021). Skeletal Aging and Osteoporosis: Mechanisms and Therapeutics. Int. J. Mol. Sci..

[47] Jiang D., Rasul A., Batool R., Sarfraz I., Hussain G., Mateen T.M., Qin T., Selamoglu Z., Ali M., Li J. (2019). Potential Anticancer Properties and Mechanisms of Action of Formononetin. Biomed. Res. Int..

[48] Li L., Wang Y., Wang X., Tao Y., Bao K., Hua Y., Jiang G., Hong M. (2018). Formononetin attenuated allergic diseases through inhibition of epithelial-derived cytokines by regulating E-cadherin. Clin. Immunol..

[49] Sun T., Liu R., Cao Y.X. (2011). Vasorelaxant and antihypertensive effects of formononetin through endothelium-dependent and -independent mechanisms. Acta Pharmacol. Sin..

[50] Yu X., Yang B., Chen B., Wu Q., Ren Z., Wang D., Yuan T., Ding H., Ding C., Liu Y. (2023). Inhibitory effects of Formononetin on CoCrMo particle-induced osteoclast activation and bone loss through downregulating NF-kappaB and MAPK signaling. Cell Signal..

[51] Jing W., Feng L., Peng K., Zhang W., Wang B. (2022). Formononetin attenuates osteoclast differentiation and calcium loss by mediating transcription factor AP-1 in type I diabetic mice. J. Biochem. Mol. Toxicol..

[52] Wu W., Fu J., Gu Y., Wei Y., Ma P., Wu J. (2020). JAK2/STAT3 regulates estrogen-related senescence of bone marrow stem cells. J. Endocrinol..

[53] Machado D.J., Espitia P., Andrade B.R. (2021). Formononetin: Biological effects and uses—A review. Food Chem..

[54] Ling Y., Nie D., Huang Y., Deng M., Liu Q., Shi J., Ouyang S., Yang Y., Deng S., Lu Z. (2023). Antioxidant Cascade Nanoenzyme Antagonize Inflammatory Pain by Modulating MAPK/p-65 Signaling Pathway. Adv. Sci..

[55] Che J., Yang X., Jin Z., Xu C. (2023). Nrf2: A promising therapeutic target in bone-related diseases. Biomed. Pharmacother..

[56] Xia Z., Dickens M., Raingeaud J., Davis R.J., Greenberg M.E. (1995). Opposing effects of ERK and JNK-p38 MAP kinases on apoptosis. Science.

[57] Jomova K., Raptova R., Alomar S.Y., Alwasel S.H., Nepovimova E., Kuca K., Valko M. (2023). Reactive oxygen species, toxicity, oxidative stress, and antioxidants: Chronic diseases and aging. Arch. Toxicol..

[58] Gong L., Lei Y., Liu Y., Tan F., Li S., Wang X., Xu M., Cai W., Du B., Xu F. (2019). Vaccarin prevents ox-LDL-induced HUVEC EndMT, inflammation and apoptosis by suppressing ROS/p38 MAPK signaling. Am. J. Transl. Res..

