# Supplementary material for: Formononetin Protects Osteoblasts Against Oxidative Stress and Attenuates Ovariectomy-Induced Bone Loss in Association with MAPK/AP-1 and NRF2 Signaling
Source: Antioxidants (Basel). 2026 Oct 19;15(10):1348. doi: 10.3390/antiox15101348 (PMC13701926; doi:10.3390/antiox15101348)
Supplement: Supplementary file 1 [file antioxidants-15-01348-s001.zip › antioxidants-4587619-supplementary.pdf]

Supporting Information

For

Formononetin protects osteoblasts against oxidative stress and attenuates ovariectomy-induced bone loss in association with MAPK/AP-1 and NRF2 signaling

This file includes:

Supplementary Figures and Figure Legends S1 to S4

Supplementary Tables S1 to S3

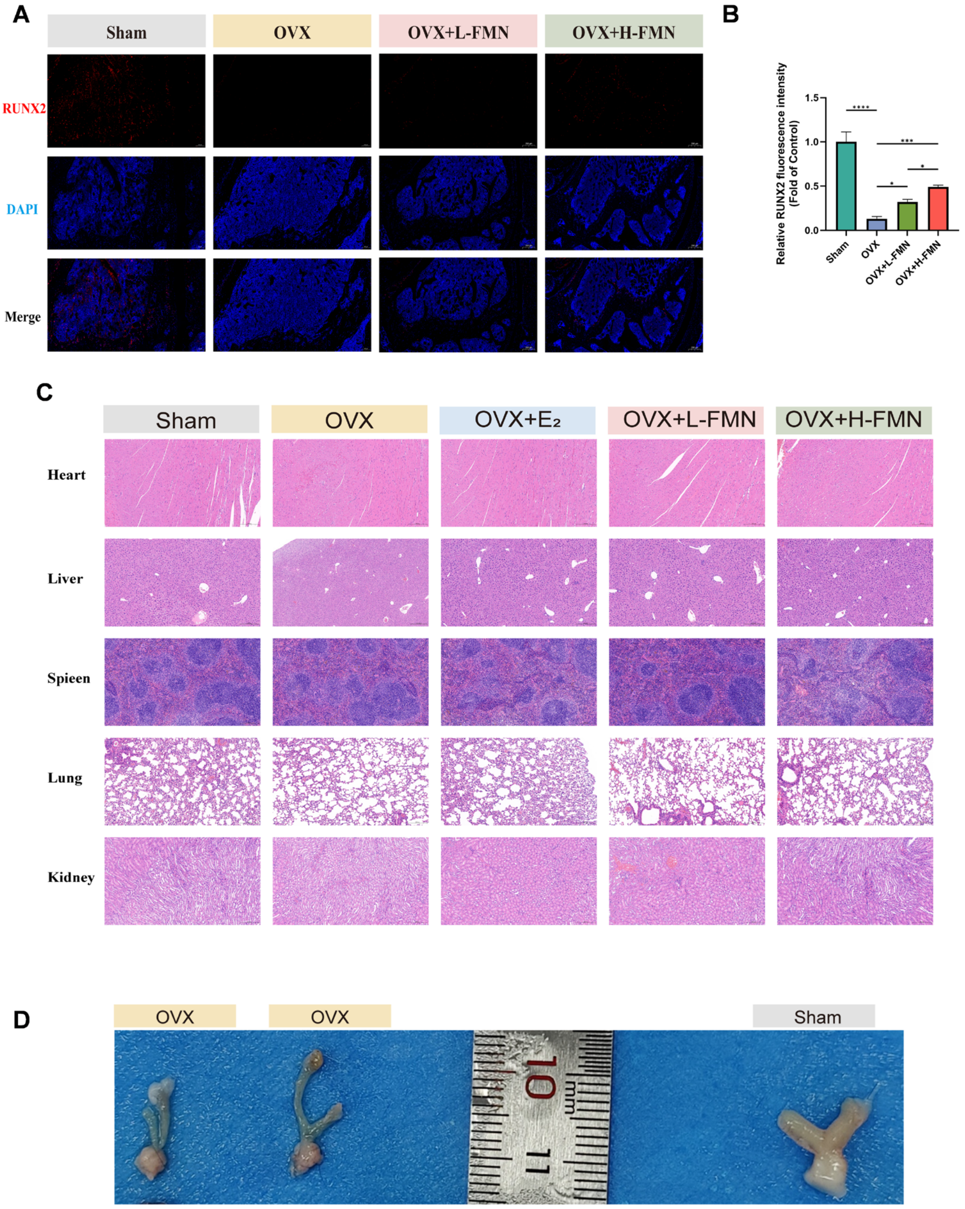

Supplementary Figure S1. FMN preserves RUNX2 expression in the distal femur and shows no obvious histological toxicity in major organs. (A) Representative RUNX2 immunofluorescence staining images of distal femoral sections. RUNX2 is shown in red, and nuclei were counterstained with DAPI in blue. (B) Quantitative analysis of RUNX2 fluorescence intensity. (C) Representative H&E staining images of the heart, liver, spleen, lung, and kidney. Scale bars = 200 μm. (D) Representative gross photographs of uterine tissues from Sham and OVX mice, showing a visible reduction in uterine size following ovariectomy. A metric ruler is included as a size reference. Data are presented as mean ± SD from three independent mice per group (n = 3). Statistical comparisons were performed using one-way ANOVA followed by Tukey's multiple-comparisons test. ns, not significant; **p* < 0.05, ****p* < 0.001, and *****p* < 0.0001.

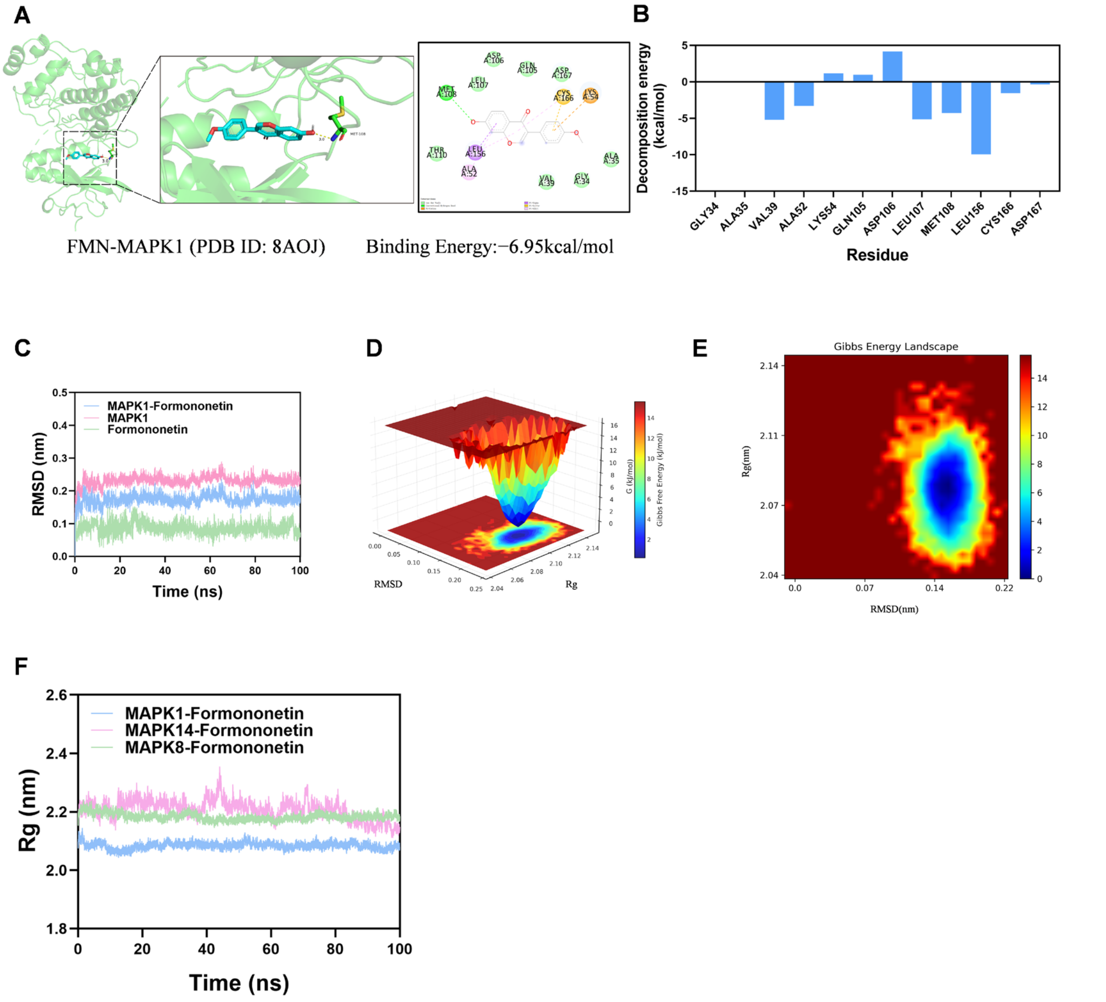

Supplementary Figure S2. Molecular docking and molecular dynamics simulation of the FMN–MAPK1 complex. (A) Molecular docking analysis of FMN with MAPK1, including the overall docking conformation, enlarged binding pocket, and two-dimensional interaction diagram. (B) Residue energy decomposition analysis of the FMN–MAPK1 complex. (C) Root mean square deviation (RMSD) plot of the FMN–MAPK1 complex during 100 ns molecular dynamics simulation. (D, E) Gibbs free energy landscape of the FMN–MAPK1 complex. (F) Radius of gyration (Rg) analysis of the FMN–MAPK1, FMN–MAPK8, and FMN–MAPK14 complexes.

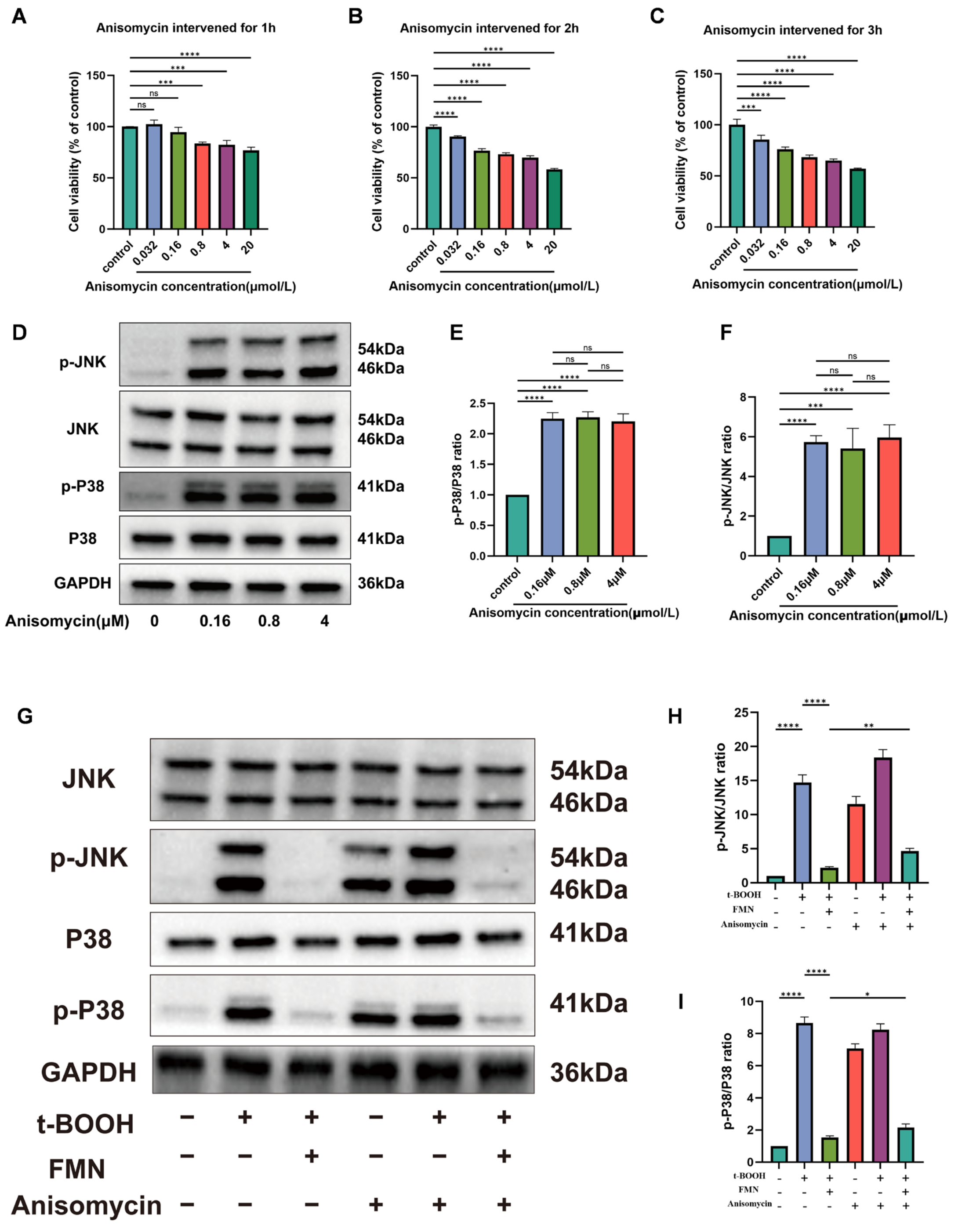

Supplementary Figure S3. Optimization and validation of anisomycin-mediated p38/JNK pathway activation. (A–C) Cell viability of MC3T3-E1 cells after treatment with anisomycin at 0.032, 0.16, 0.8, 4, and 20 μM for 1 h (A), 2 h (B), and 3 h (C), respectively. (D) Representative western blot analysis of p-JNK, JNK, p-p38, and p38 after treatment with anisomycin at 0.16, 0.8, and 4 μM for 1 h, with GAPDH shown as the loading control. (E, F) Quantitative analysis of the p-p38/p38 ratio (E) and p-JNK/JNK ratio (F). (G) Representative western blot analysis of p-JNK, JNK, p-p38, and p38 under the indicated t-BOOH, FMN, and anisomycin treatment conditions. (H, I) Quantitative analysis of the p-JNK/JNK ratio (H) and

p-p38/p38 ratio (I). Data are presented as mean ± SD from three independent biological experiments (n = 3). Statistical comparisons were performed using one-way ANOVA followed by Tukey's multiple-comparisons test. ns, not significant; *$p < 0.05$, **$p < 0.01$, ***$p < 0.001$, and ****$p < 0.0001$.

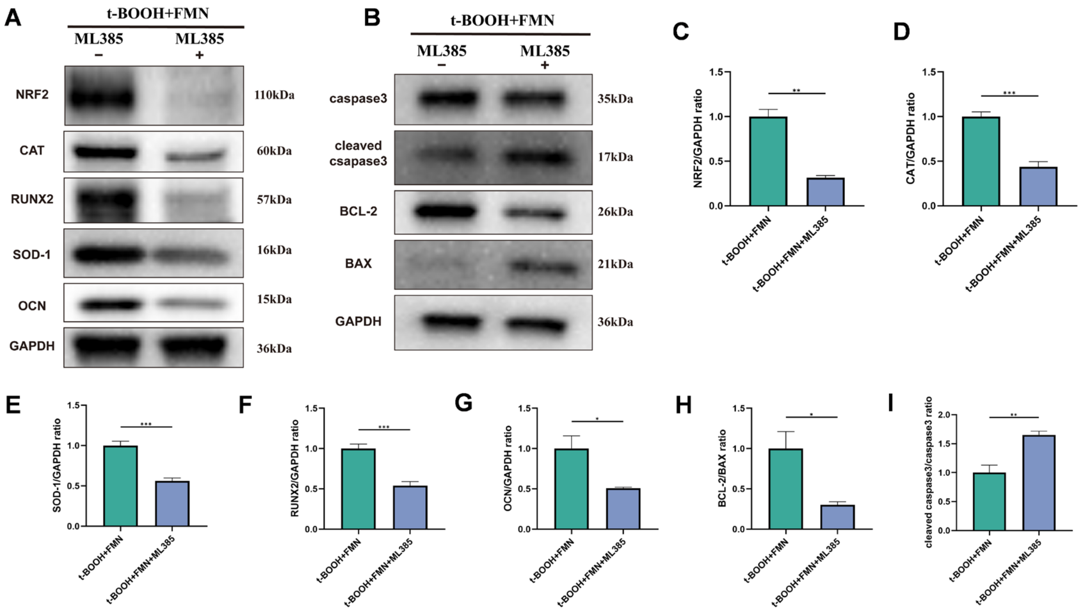

Supplementary Figure S4. NRF2 inhibition attenuates the antioxidant, anti-apoptotic, and pro-osteogenic effects of FMN under oxidative stress. (A) Western blot analysis of NRF2, CAT, RUNX2, SOD1, and OCN in t-BOOH+FMN-treated MC3T3-E1 cells with or without ML385, using GAPDH as the internal reference. (B) Western blot analysis of caspase-3, cleaved caspase-3, BCL-2, and BAX using GAPDH as the internal reference. (C–G) Quantitative analysis of NRF2 (C), CAT (D), SOD1 (E), RUNX2 (F), and OCN (G) protein levels. (H, I) Quantitative analysis of BCL-2/BAX ratio (H) and cleaved caspase-3/caspase-3 ratio (I). Data are presented as mean ± SD from three independent biological experiments (n = 3). Statistical comparisons were performed using an unpaired two-tailed Student's t-test. ns, not significant; *$p < 0.05$, **$p < 0.01$, and ***$p < 0.001$. ML385, NRF2 inhibitor.

Supplementary Table S1. The target of FMN (n = 166).

Target	Target	Target	Target	Target	Target
ATP5F1B	HSD3B1	HSD3B2	IL4	JUN	SIRT1
DPEP1	ADRA1D	PDE3A	PIM1	CYP19A1	ALDH2
CA12	ADORA1	ADORA2A	TBXAS1	MAOA	MGAM
HTR2A	HTR2C	ESRRB	ABCG2	CA7	HSD17B2
CA4	CBR1	SLC6A2	ALOX12	EGFR	TYR
XDH	PFKFB3	PTPRS	HSD17B1	MIF	ALOX15
PPARA	TLR9	CA1	NOX4	PON1	TNKS2
TNKS	CA3	CA6	CA14	CA13	CA5B
CA5A	DHODH	STS	ERCC5	FEN1	RAF1
CA9	PTPN1	BACE1	TYK2	EZR	BAD
IKBKE	MCL1	ALOX5	AVPR1A	PLA2G4A	TNF
GRK6	AKR1B1	ABL1	HCK	PI4KB	HSP90AB1
EPHB4	KIT	OPRD1	PTK6	KIF11	KDM5C
KDM4A	KDM4D	KDM4C	CNOT7	AURKB	LIG1
BIRC5	KCNMA1	FLT3	LRRK2	GCGR	PTGER1
PTGER2	PTGER3	PTGDR	PLG	CCNB3	CDK1
CCNB2	IL2	ESRRA	ABCB1	BCL2	CYP1B1
HSP90AA1	CCNB1	ADRA1A	ACHE	MAOB	CA2
CASP9	NFE2L2	SMN2	UGT1A7	ATAD5	ABCC2
CYP1A2	HIF1A	UGT1A1	NFKBIA	MAPT	TP53
HDAC9	UGT1A8	CXCL8	CYP1A1	RARA	SMN1
YAP1	PRKACA	CYP2D6	NR1I2	NR5A1	CASP3
HSD17B10	UGT1A9	NR1I3	CASP8	EPO	ABCC1
CCND1	UGT1A10	BAX	GLI3	NR0B1	AHR
PGR	PTGS1	ESR1	PPARG	ESR2	GSK3B
CHEK1	CDK2	MAPK14	DRD1	PTGS2	RXRA
ADRB2	CCNA2	PKIA	DPP4	SLC6A3	NOS2
SLC6A4	CHRM1	AR	PRSS1		

Note: Targets were integrated from TCMSP, HERB, SwissTargetPrediction, and ITCM; duplicates were removed.

Supplementary Table S2. Drug–disease intersection targets (n = 139).

Target	Target	Target	Target	Target
HSD3B1	HSD3B2	IL4	JUN	SIRT1
PDE3A	CYP19A1	ALDH2	ADORA1	ADORA2A
TBXAS1	MAOA	MGAM	HTR2A	HTR2C
ESRRB	ABCG2	CA4	SLC6A2	ALOX12
EGFR	TYR	XDH	PFKFB3	PTPRS
MIF	ALOX15	PPARA	TLR9	CA1
NOX4	PON1	TNKS	CA6	CA5A
DHODH	STS	ERCC5	RAF1	CA9
PTPN1	BACE1	TYK2	EZR	BAD
IKBKE	MCL1	ALOX5	AVPR1A	PLA2G4A
TNF	AKR1B1	ABL1	PI4KB	HSP90AB1
KIT	OPRD1	KIF11	KDM5C	KDM4C
AURKB	LIG1	BIRC5	KCNMA1	FLT3
LRRK2	GCGR	PTGER1	PTGER2	PTGER3
PTGDR	PLG	CDK1	IL2	ESRRA
ABCB1	BCL2	CYP1B1	HSP90AA1	CCNB1
ADRA1A	ACHE	MAOB	CA2	CASP9
NFE2L2	SMN2	UGT1A7	ABCC2	CYP1A2
HIF1A	UGT1A1	NFKBIA	MAPT	TP53
HDAC9	UGT1A8	CXCL8	CYP1A1	RARA
SMN1	YAP1	PRKACA	CYP2D6	NR1I2
NR5A1	CASP3	HSD17B10	UGT1A9	NR1I3
CASP8	EPO	ABCC1	CCND1	UGT1A10
BAX	GLI3	NR0B1	AHR	PGR
PTGS1	ESR1	PPARG	ESR2	GSK3B
CHEK1	CDK2	MAPK14	PTGS2	RXRA
ADRB2	CCNA2	PKIA	DPP4	SLC6A3
NOS2	SLC6A4	AR	PRSS1	

Note: Osteoporosis-related targets were collected from GeneCards, DisGeNET, and OMIM. The table lists the 139 unique targets shared between the FMN-related and osteoporosis-related target sets.

Supplementary Table S3. Molecular docking parameters for the FMN–MAPK complexes.

Protein	Grid center X (Å)	Grid center Y (Å)	Grid center Z (Å)	Grid size X (Å)	Grid size Y (Å)	Grid size Z (Å)	Lowest predicted binding energy (kcal/mol)
MAPK1	-4.415	4.306	39.145	63.75	47.25	71.25	-6.949
MAPK8	16.382	15.079	28.018	47.25	56.25	71.25	-7.965
MAPK14	11.141	-4.721	-16.192	75	47.25	52.5	-8.763

Supplementary Table S3. Molecular docking parameters for the FMN–MAPK complexes. The table summarizes the docking search-space settings used for AutoDock Vina analysis of formononetin (FMN) against MAPK1 (PDB ID: 8AOJ), MAPK8 (PDB ID: 4QTD), and MAPK14 (PDB ID: 3LFF). Grid center coordinates and box dimensions are reported in Å. A grid spacing of 0.375 Å and an exhaustiveness value of 8 were used for all docking runs. The lowest predicted binding energy of the top-ranked docking pose is shown for each protein.
